# Large-area synthesis of high-quality and uniform monolayer WS_2_ on reusable Au foils

**DOI:** 10.1038/ncomms9569

**Published:** 2015-10-09

**Authors:** Yang Gao, Zhibo Liu, Dong-Ming Sun, Le Huang, Lai-Peng Ma, Li-Chang Yin, Teng Ma, Zhiyong Zhang, Xiu-Liang Ma, Lian-Mao Peng, Hui-Ming Cheng, Wencai Ren

**Affiliations:** 1 Shenyang National Laboratory for Materials Science, Institute of Metal Research, Chinese Academy of Sciences, 72 Wenhua Road, Shenyang 110016, China; 2 Key Laboratory for the Physics and Chemistry of Nanodevices, Department of Electronics, Peking University, Beijing 100871, China

## Abstract

Large-area monolayer WS_2_ is a desirable material for applications in next-generation electronics and optoelectronics. However, the chemical vapour deposition (CVD) with rigid and inert substrates for large-area sample growth suffers from a non-uniform number of layers, small domain size and many defects, and is not compatible with the fabrication process of flexible devices. Here we report the self-limited catalytic surface growth of uniform monolayer WS_2_ single crystals of millimetre size and large-area films by ambient-pressure CVD on Au. The weak interaction between the WS_2_ and Au enables the intact transfer of the monolayers to arbitrary substrates using the electrochemical bubbling method without sacrificing Au. The WS_2_ shows high crystal quality and optical and electrical properties comparable or superior to mechanically exfoliated samples. We also demonstrate the roll-to-roll/bubbling production of large-area flexible films of uniform monolayer, double-layer WS_2_ and WS_2_/graphene heterostructures, and batch fabrication of large-area flexible monolayer WS_2_ film transistor arrays.

## Abstract (web summary)

WS_2_ is a promising material for application in next-generation electronics, yet current methods of fabrication can only yield micrometre-sized domains. Here, via ambient-pressure CVD, the authors report the growth of high-quality, uniform monolayer WS_2_ single crystals of the order of millimetres.

## Graphical abstract

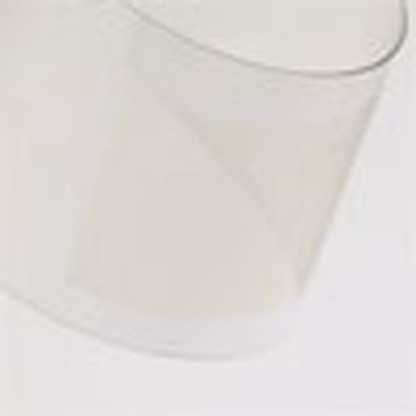

Atomically thin two-dimensional (2D) transition metal dichalcogenides (TMDCs) have attracted increasing interest because of their unique electronic and optical properties that are distinct from and complementary to graphene^1^,^2^,^3^. Different from multilayers, monolayer semiconducting TMDCs have sizable direct bandgaps^1^,^2^,^3^ and show efficient valley polarization by optical pumping^4^,^5^,^6^, which opens up the possibility for their use in transistors^7^,^8^,^9^,^10^, integrated circuits^11^, photodetectors^12^, electroluminescent devices^13^ and valleytronic and spintronic devices^5^,^6^. Besides the number of layers, defects^14^,^15^ and grain boundaries^9^ in 2D TMDCs also have a strong influence on their electronic and optical properties. Therefore, similar to current silicon-based electronics, the availability of wafer-size, defect-free and uniform monolayer TMDC single crystals is necessary for their future electronic, optoelectronic and spintronic applications.

Monolayer WS_2_ is a very important example of 2D TMDCs with a direct bandgap, which exhibits many fascinating properties and promises applications in electronics and optoelectronics^1^,^2^,^3^,^4^,^16^,^17^. It is a robust platform to study spin and valley physics because of its large spin–orbit interaction^4^. Vertical field-effect transistors (FETs) on the basis of graphene/WS_2_ heterostructures show an unprecedented high ON/OFF ratio and a very large ON current because of a combination of tunnelling and thermionic transport^16^. Chemical vapour deposition (CVD) on inert substrates such as SiO_2_/Si (refs 7, 18, 19, 20, 21, 22, 23) has shown a potential to grow large-area WS_2_ compared with other methods such as mechanical cleavage^24^,^25^ and liquid exfoliation^3^,^26^. However, as with the growth of graphene on the SiO_2_/Si substrate^27^, the material obtained suffers from unsatisfactory structure control and low quality^7^,^18^,^19^,^20^,^21^. The growth of a uniform monolayer of WS_2_ over a large area has not yet been achieved, and the domain size of the crystals is usually smaller than 100 μm (refs 7, 18, 19, 21). More importantly, many structural defects exist in 2D TMDCs CVD-grown on inert substrates, including sulphur vacancies, antisite defects, adatoms and dislocations^14^,^15^. Such defects introduce localized states in the bandgap, leading to hopping transport behaviour and consequently a dramatic decrease in the carrier mobility^15^. Monolayer WS_2_ CVD-grown on SiO_2_/Si substrates shows a very low carrier mobility of ∼0.01 cm^2 ^V^−1 ^s^−1^ at room temperature^7^, far below that of mechanically exfoliated samples (∼10 cm^2 ^V^−1 ^s^−1^) measured under similar conditions^24^.

In addition to conventional rigid device applications, transparent and flexible electronic and optoelectronic devices have been widely considered to be one of the most appealing applications of all 2D materials because of their high transparency and good flexibility^1^,^16^. To this end, the low-cost fabrication of large-area monolayer WS_2_ films on flexible substrates is highly desired. For CVD-grown 2D WS_2_ materials on inert substrates, however, the growth substrates usually have to be etched away by acidic or basic solutions such as concentrated HF, KOH and NaOH before they are transferred to flexible substrates^7^,^18^,^19^,^20^,^21^. This transfer method not only leads to inevitable damage to the WS_2_ but also produces substrate residues and serious environmental pollution, and increases production cost. Moreover, the rigid nature of inert substrates is not compatible with the cost- and time-effective roll-to-roll production technique, which has been used for the fabrication of large-area graphene-transparent conductive films as well as of high-throughput printed flexible electronics^28^,^29^.

Here we develop a scalable ambient-pressure catalytic CVD method on the basis of the self-limited surface growth mechanism, using flexible Au foils as substrate, to grow high-quality uniform monolayer WS_2_ single crystals of the order of millimetres and large-area continuous films. These WS_2_ monolayers can be transferred from Au foils to arbitrary substrates without damage to either the WS_2_ or the Au foils by using an electrochemical bubbling method because of the weak interaction between monolayer WS_2_ and the Au substrate in this unique growth process. The WS_2_ domains show optical and electrical properties comparable or superior to those of mechanically exfoliated samples, and their carrier mobility and ON/OFF ratio are 100 times higher than those of CVD-grown samples on SiO_2_/Si substrates measured under similar conditions^7^. Using the good flexibility of Au foils and the electrochemical bubbling method, we also demonstrate the roll-to-roll/bubbling production of large-area, transparent, flexible monolayer WS_2_ films and their heterostructures with graphene films without sacrificing the Au foils, and batch fabrication of large-area flexible monolayer WS_2_ film transistor arrays with electrical performance comparable to those on SiO_2_/Si substrates.

## Results

### Growth and transfer of uniform monolayer WS_2_

**Figure 1:**
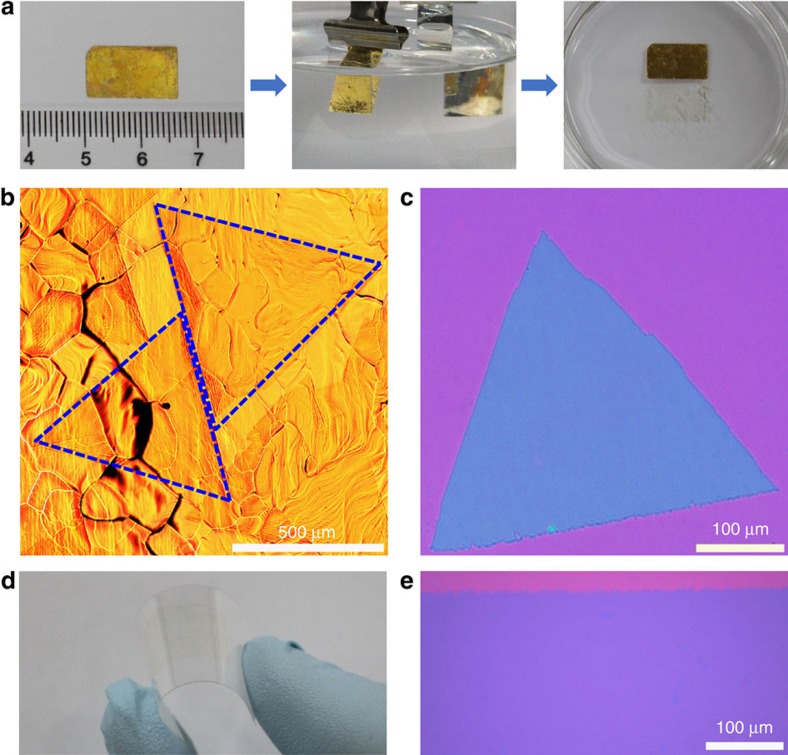
CVD growth of monolayer WS_2_ single crystals and films on Au foils and electrochemical bubbling transfer. (**a**) Electrochemical bubbling transfer of the monolayer WS_2_ grown on a Au foil by ambient-pressure CVD. Left panel, the Au foil with monolayer WS_2_ covered by a polymethyl methacrylate (PMMA) layer. Middle panel, the PMMA/monolayer WS_2_ was gradually separated from the Au foil driven by the H_2_ bubbles produced at the cathode when applying a constant current. Right panel, the completely separated PMMA/monolayer WS_2_ and Au foil after bubbling for 30 s, showing the intact Au foil. (**b**) Optical image of millimetre-sized triangular monolayer single-crystal WS_2_ domains on the Au foil. (**c**) Optical image of a 400-μm single-crystal WS_2_ domain transferred on a SiO_2_/Si substrate. (**d**) Photograph of a monolayer WS_2_ film transferred on a PET substrate, showing good flexibility. (**e**) Optical image of the edge region of a monolayer WS_2_ film transferred on a SiO_2_/Si substrate, showing uniform thickness without any additional layers and visible cracks.

As described in Methods (Fig. 1 and Supplementary Fig. 1), we grew monolayer WS_2_ single crystals and films on the Au foil by ambient-pressure CVD at 800 °C using WO_3_ and sulphur powders as precursors. The reasons why we chose Au as the growth substrate are as follows: Au is the only metal that does not form any sulphide when reacting with sulphur at high temperature (500 °C or even higher); Au is a commonly used catalyst for the growth of different materials including graphene and carbon nanotubes^30^,^31^,^32^, and it can lower the barrier energies for the sulphurization of WO_3_ through the formation of sulphur atoms (Supplementary Fig. 2 and Supplementary Fig. 3), which allows the use of a very low concentration of WO_3_ and sulphur and thus is beneficial for the formation of monolayer WS_2_ with much higher quality than would be produced using inert substrates; and the solubility of W in Au is extremely low (below 0.1 atomic% at 800 °C), which makes the segregation and precipitation processes for multilayer growth impossible^33^. These three features make it possible to grow uniform high-quality monolayer WS_2_ on Au by a self-limited surface-catalytic process similar to that used for the growth of graphene on Cu (ref. 34). In addition, the good flexibility of the Au foil is compatible with the large-area roll-to-roll production process^28^.

As shown in Fig. 1 and Supplementary Fig. 4, all the isolated WS_2_ domains have a regular triangular shape, a typical characteristic of single-crystal WS_2_ prepared by CVD^7^,^18^,^19^,^21^. Atomic force microscope (AFM) measurements show that the thickness of the WS_2_ obtained is ∼0.95 nm (Supplementary Fig. 5), indicating that they are monolayers. Moreover, the domain size is increased by increasing the growth time, lowering the heating temperature of the sulphur powder and increasing the Ar flow rate in the reaction zone (Supplementary Fig. 4 and Supplementary Fig. 6). Under optimized conditions, millimetre-size single-crystal monolayer WS_2_ domains were obtained (Fig. 1b), and these are much larger than the samples CVD-grown on inert substrates and mechanically or liquid-exfoliated samples^7^,^18^,^19^,^21^,^24^,^26^, which are typically smaller than 100 μm. It is needed to point out that all the WS_2_ domains obtained under the above conditions are monolayers despite their different domain size, indicating a broad window for monolayer WS_2_ growth on Au. By increasing the growth time, adjacent domains expand, join and eventually form a continuous monolayer WS_2_ film (Supplementary Fig. 7), a typical characteristic of surface growth. It is important to note that no additional layers were formed on the film even by further increasing the growth time (Supplementary Fig. 7). Moreover, there were still enough WO_3_ and sulphur source for the growth of WS_2_ in the reaction system after the growth of a monolayer WS_2_ film, which was confirmed by the growth of monolayer WS_2_ domains on a newly loaded bare Au foil (Supplementary Fig. 8). These facts confirm the self-limited surface growth behaviour of monolayer WS_2_ on Au substrates.

We also compared the growth of WS_2_ on the Au foil and the commonly used SiO_2_/Si substrate under the same experimental conditions. It is worth noting that no WS_2_ domains were formed on the SiO_2_/Si substrate when we used the same growth conditions as those for WS_2_ growth on Au (Supplementary Fig. 9). However, a few WS_2_ domains can be formed if the concentration of sulphur in the reaction system was greatly increased (the evaporation area of sulphur powder was increased by ∼10 times, and its heating temperature was increased by 30 °C; Supplementary Fig. 10a). In contrast, many multilayer WS_2_ domains were formed on the Au foil under the same conditions (Supplementary Fig. 10b and Supplementary Note 1). These results give strong evidence that the Au helps the growth of WS_2_, which is consistent with the barrier energy decrease for the sulphurization of WO_3_ by sulphur atoms on Au compared with the direct sulphurization of WO_3_ in sulphur atmosphere based on density functional theory (DFT) calculations (Supplementary Fig. 2 and Supplementary Fig. 3). Therefore, a low concentration of WO_3_ and sulphur that is insufficient for spontaneously sulphurization reaction can be used for WS_2_ growth on Au. Actually, it has been demonstrated that a very low concentration of precursors is essential for growing monolayer graphene^35^. Although Cu has a very low solubility for carbon, increasing the concentration of methane in the reaction system can lead to the formation of bi-, tri- and tetra-layer graphene films^35^. This is also the reason why multilayer WS_2_ was formed on the Au foil when the concentration of sulphur in the reaction system was greatly increased (Supplementary Fig. 10b). It is worth noting that, when the Au substrate was fully covered by WS_2_ film, the formation of sulphur atoms and the sulphurization of WO_3_ were suppressed, and consequently no additional layers were formed even further extending the growth time under the same conditions as those for WS_2_ growth on Au. Therefore, the barrier energy decrease for the sulphurization of WO_3_ on Au compared with that in sulphur atmosphere plays an important role for the self-limited growth of monolayer WS_2_ on Au.

Actually, Au has also been reported as a substrate for the growth of monolayer to few-layer MoS_2_ (refs 36, 37, 38, 39, 40). However, it is worth noting that the solubility of Mo in Au (∼0.34 atomic% at 300 °C and ∼0.9 atomic% at 800 °C) is significantly higher than that of W (below 0.1 atomic% at 800 °C), which makes the growth of MoS_2_ on Au and its subsequent transfer completely different from those of the monolayer WS_2_ reported here. During the two-step growth process of MoS_2_ on the Au substrate, it has been confirmed that Mo–Au surface alloys are first formed and then transformed to MoS_2_ on exposure to H_2_S. In this case, only few-layer MoS_2_ was obtained by ambient-pressure CVD, and there are still a certain portion of Mo–Au alloys left after the formation of MoS_2_. In contrast, no WS_2_ domains were formed when the Au substrate first reacted with WO_3_ without the presence of sulphur and then the obtained substrate reacted with sulphur without the presence of WO_3_ (Supplementary Fig. 11), and no W–Au alloy signals were detected after the growth of monolayer WS_2_ (Supplementary Fig. 12). On the basis of the above analyses, we suggest that the growth of MoS_2_ on Au (ref. 39) is similar to the growth of graphene on Ni (ref. 41), and it is difficult to control the number of layers of MoS_2_ by ambient-pressure CVD^39^. Two to three layers of MoS_2_ with a very low carrier mobility of ∼0.004 cm^2 ^V^−1 ^s^−1^ were obtained in this case^39^. To grow monolayer MoS_2_ on the Au substrate, ultrahigh vacuum deposition^39^ or low-pressure CVD^36^,^37^,^38^ has to be used to reduce the supply of Mo and S for the deposition reaction, similar to the growth of monolayer graphene on Ni (ref. 42). In contrast, similar to the graphene growth on Cu (ref. 34), the low solubility of tungsten in Au makes the segregation and precipitation processes for multilayer growth impossible, which is another important reason for the self-limited growth of monolayer WS_2_ on Au substrates. In this catalytic surface growth process, the passivation of surface imperfections in Au foils by polishing and annealing before CVD growth play an important role in the growth of millimetre-size WS_2_ domains (Supplementary Fig. 13 and refs 43, 44).

The weak interaction between the monolayer WS_2_ and the Au substrate is another very important feature of the samples prepared by ambient-pressure CVD method (Supplementary Fig. 14). We can also obtain monolayer WS_2_ on a Au foil by low-pressure CVD (Supplementary Fig. 15); however, the monolayers MoS_2_ and WS_2_ grown by low-pressure CVD have a strong interaction with the Au substrate (Supplementary Fig. 14 and refs 36, 37, 38). As a result, the 2D TMDC cannot be separated from the Au substrate by a nondestructive bubbling method (Supplementary Fig. 15) and have to be transferred by etching away the Au substrate^36^,^37^,^38^,^39^. This makes the fabrication of monolayers MoS_2_ and WS_2_ on the Au substrate by low-pressure CVD too expensive to be used for large-scale production. We also studied the electrochemical bubbling transfer of the WS_2_ samples grown by ambient-pressure CVD followed by low-pressure CVD-successive growth or annealing (Supplementary Fig. 16). It is interesting to find that the ambient-pressure CVD-grown samples cannot be separated by bubbling any more once they are subjected to low-pressure treatments. Moreover, we can clearly see the contrast and roughness difference between the regions grown under ambient-pressure and low-pressure CVD condition in the same domain. These indicate that there are probably some gases trapped between the 2D TMDC and the Au substrate in ambient-pressure CVD process, and these gases tend to be expelled under low-pressure conditions, which lead to remarkable difference in the interaction between the 2D TMDC and the Au substrate for the samples prepared by ambient-pressure and low-pressure CVD processes.

**Figure 2:**
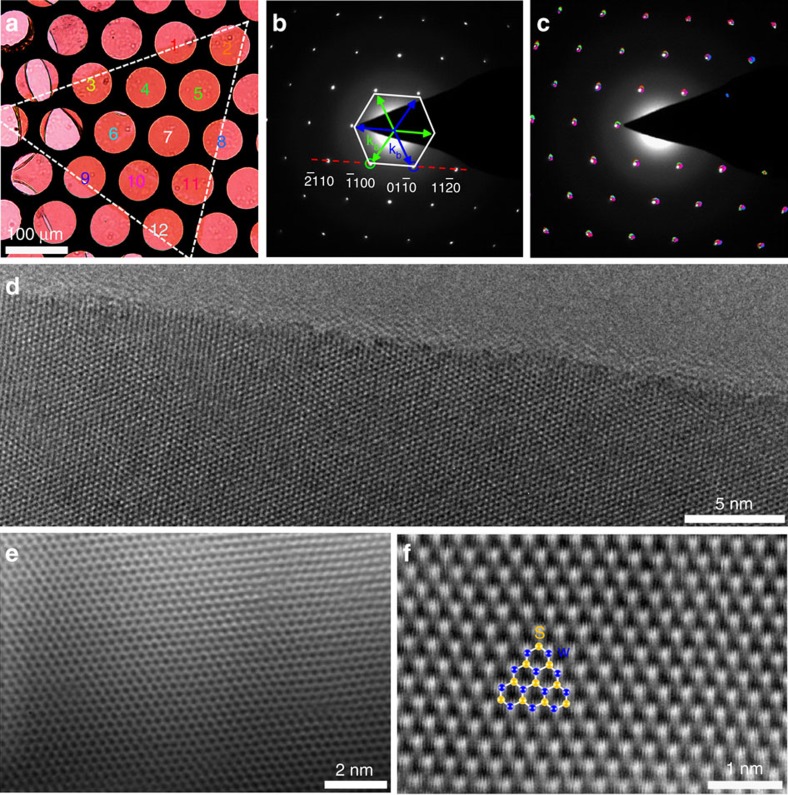
Structural characterization of monolayer single-crystal WS_2_ domains. (**a**) Optical image of a 400-μm monolayer WS_2_ domain transferred on a TEM grid. (**b**) Typical SAED pattern of the sample in **a** showing a hexagonal crystal structure. The asymmetry of the W and S sublattices separates the 
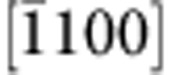
 diffraction spots into two families: 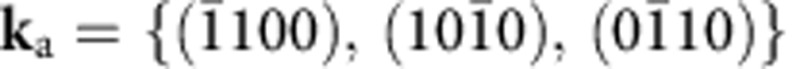 and **k**_b_=−**k**_a_. (**c**) Superimposed image of 12 coloured SAED patterns taken from the areas labelled with numbers 1–12 in **a**. The same colours were used to indicate the SAED patterns and the corresponding areas in **a**, where they were collected. (**d**–**f**) HRTEM (**d**) aberration-corrected HRTEM, (**e**) aberration-corrected ADF-STEM (**f**) images of the monolayer WS_2_ domains, showing no point defects, voids and sulphur vacancies.

The weak interaction between the monolayer WS_2_ and the Au substrate for our samples enables the intact transfer of WS_2_ from the substrate using the electrochemical bubbling method^45^. After CVD growth, therefore, we used an electrochemical bubbling method on the basis of the water electrolysis reaction^45^ to transfer the monolayer WS_2_ from Au foils to SiO_2_/Si substrates (the thickness of the SiO_2_ layer is 290 nm), polyethylene terephthalate (PET) and transmission electron microscopy (TEM) grids for further characterizations (Figs 1a,c–e and 2a). Notably, all the transferred domains perfectly preserved their original structure on the Au substrates (Supplementary Fig. 17), and no optical contrast difference was found for all domains and within a single domain on SiO_2_/Si substrates (Fig. 1c,e), which indicates the highly uniform thickness of the sample. The Au substrate is chemically inert to the electrolyte used and was not involved in the water electrolysis reaction during transfer. As a result, it was not destroyed and could be used repeatedly (Fig. 1a, Supplementary Fig. 12 and Supplementary Fig. 18). So far, one Au foil has been reused ∼700 times, and most of the Au foils have been reused more than 300 times. Moreover, the monolayer WS_2_ domains grown on reused Au foils showed a similar structure to those grown on the original ones (Supplementary Fig. 19). The nondestructive transfer makes the CVD method using Au as a substrate suitable for the scalable production of monolayer WS_2_ at a low cost.

### Structure and optical/electrical properties

We used selected-area electron diffraction (SAED), high-resolution TEM (HRTEM) and atomic resolution scanning TEM (STEM) to characterize the crystal structure of the large WS_2_ domains (Fig. 2). Figure 2a shows an optical image of a 400-μm WS_2_ domain transferred on a TEM grid. Similar to monolayer MoS_2_ (ref. 9), monolayer WS_2_ shows a hexagonal structure with threefold symmetry in terms of the two sets of spots in its diffraction pattern (Fig. 2b,c and Supplementary Fig. 20), bright **k**_**a**_ spots (indicated by green arrows) and dark **k**_**b**_ spots (indicated by blue arrows). To identify whether this domain is a single crystal, we carried out a series of SAED measurements on 12 different regions by carefully moving the sample without rotation or tilting. Figure 2c is a superimposed image of the 12 SAED patterns. The perfect coincidence of all the diffraction patterns confirms the single-crystal nature of this large WS_2_ domain. Similar measurements and analyses of dozens of samples show that all the triangular WS_2_ domains are single crystals.

Figure 2d,e shows representative HRTEM and aberration-corrected HRTEM images of our single-crystal WS_2_ sample, respectively. No point defects or voids are observed over a large area. Moreover, in addition to the commonly observed zigzag edges (Supplementary Fig. 21), we also find that some monolayer WS_2_ single crystals have an edge along armchair crystallographic orientation (Fig. 2d, Supplementary Fig. 22 and Supplementary Fig. 23). This is different from the triangular WS_2_ or MoS_2_ single crystals grown on the SiO_2_/Si substrate, in which only zigzag edges have been observed^9^,^20^. Actually, different edges have also been observed in the graphene grown on chemically inert SiC substrate (armchair edge; ref. 46) and metal substrates such as Cu and Pt (zigzag edge; refs 47, 48). We suggest that this edge difference is probably attributed to the different interfacial interactions for 2D materials grown on metals and inert substrates or on different faces of the same metal substrate. Further experimental studies combined with theoretical calculations are required to elucidate this point in the future.

We then used an aberration-corrected annular dark-field STEM (ADF-STEM), operated at a low voltage of 60 keV, to further identify the atomic structure of our WS_2_ samples. The ADF-STEM image intensity scales roughly as the square of the atomic number of the atom, allowing atom-by-atom structural and chemical identification by quantitative analysis of the image intensity. Figure 2f shows a representative ADF-STEM image of our WS_2_, which shows a clear hexagonal lattice structure with threefold symmetry. The bright spots correspond to tungsten atoms and the dim spots correspond to two stacked sulphur atoms. The significant difference in image intensity is attributed to the big difference between the atomic numbers of W and S. The uniform intensity difference between the bright and dim spots indicates that there are no sulphur vacancies (Fig. 2f).

**Figure 3:**
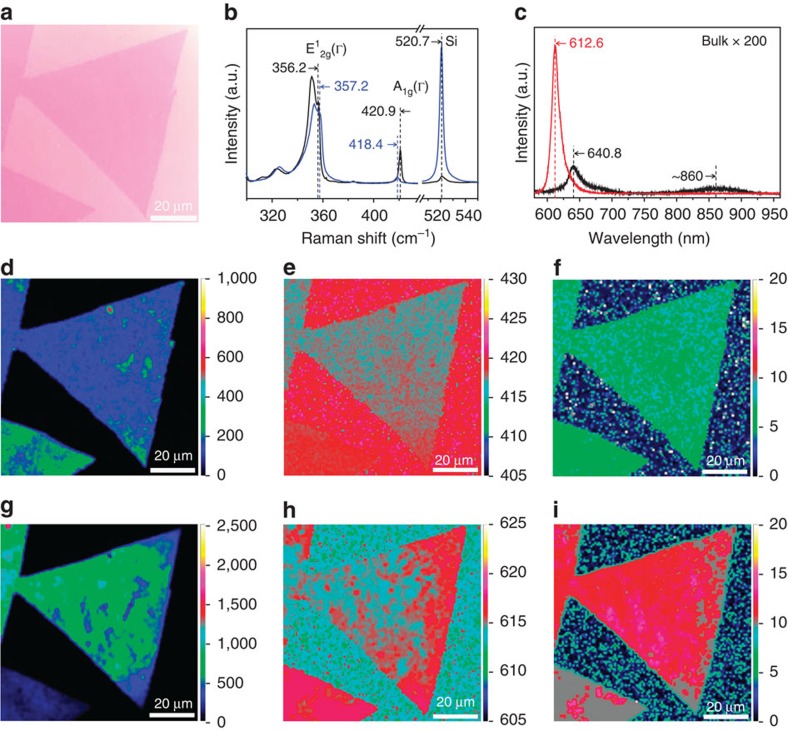
Optical properties of monolayer single-crystal WS_2_ domains. (**a**) Optical image of an ∼100-μm monolayer WS_2_ domain transferred on a SiO_2_/Si substrate. (**b**) Typical Raman spectra of the monolayer WS_2_ (blue plot) in **a** and bulk *H*-WS_2_ (black plot). (**c**) Typical PL spectra of the monolayer WS_2_ (red plot) in **a** and bulk *H*-WS_2_ (black plot). (**d**–**f**) Raman maps of the intensity (**d**), position (**e**) and full-width at half-maximum (FWHM; **f**) of *A*_1g_ peak of the monolayer WS_2_ domain in **a**. (**g**–**i**) PL maps of the intensity (**g**), position (**h**) and FWHM (**i**) of ∼613-nm PL peak of the monolayer WS_2_ domain in **a**.

We further used Raman and photoluminescence (PL) spectroscopy to characterize the structure, quality and optical properties of the large WS_2_ domains. Figure 3a–c shows an optical image and the corresponding Raman and PL spectra of an ∼100-μm triangular WS_2_ domain on the SiO_2_/Si substrate. The Raman spectrum shows typical features of monolayer WS_2_: the *E*^1^_2g_ and *A*_1g_ modes are, respectively, located at ∼357 and 418 cm^−1^, representing an upward shift of ∼1 cm^−1^ and a downward shift of ∼3 cm^−1^ compared with those of bulk WS_2_; the *E*^1^_2g_ and 2LA (M) modes are much stronger than the *A*_1g_ mode; and the absence of the peak at ∼310 cm^−1^ (ref. 49). In addition, it shows a very sharp and strong single PL peak at ∼612.6 nm (2.02 eV) in the range of 575–975 nm, which represents an upward shift of ∼0.09 eV and is much narrower and ∼10^3^ times stronger than the asymmetric peak at ∼641 nm of bulk WS_2_. The strong single PL peak indicates that the WS_2_ is a direct-gap semiconductor with only one direct electronic transition, further confirming that our WS_2_ samples grown on Au foils are monolayer. Figure 3d–i shows the intensity, peak position and full-width at half-maximum maps of the Raman and PL spectra of the single-crystal monolayer WS_2_ in Fig. 3a. It is worth noting that all the maps are very uniform across the whole domain, indicating the high uniformity in the number of layers and crystalline quality of the sample. In addition, the full-width at half-maximum of the PL peak of our samples (33–46 meV) is much narrower than for monolayer WS_2_ grown by CVD on the SiO_2_/Si substrate (42–68 meV; ref. 19), further confirming the higher crystalline quality of our samples. Considering the difficulty in laser focusing and time consuming when mapping over a large area, the uniformity of much larger WS_2_ samples was confirmed by the uniform Raman spectra randomly taken from many positions in a single domain (Supplementary Fig. 24).

**Figure 4:**
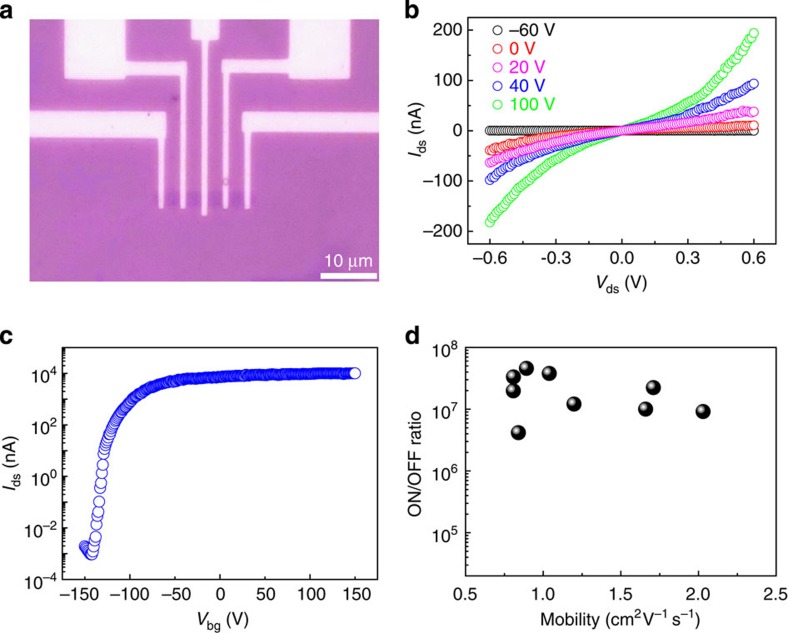
Electrical properties of monolayer single-crystal WS_2_ domains. (**a**) Optical image of a monolayer WS_2_ BG-FET. (**b**) Room-temperature output characteristics of the BG-FET. (**c**) Logarithmic transfer characteristics of the BG-FET with 5-V applied bias voltage *V*_ds_. (**d**) A summary of the room-temperature carrier mobility and the corresponding current ON/OFF ratio of nine monolayer WS_2_ BG-FETs.

To evaluate the electrical quality of our monolayer WS_2_, we fabricated back-gate FETs (BG-FETs) with transferred WS_2_ single crystals on SiO_2_/Si substrates. Both channel length and width are 3 μm in all BG-FETs (Fig. 4a). The devices were measured in vacuum at room temperature. Figure 4c shows the typical electrical transport of a monolayer WS_2_ BG-FET, indicating that the transferred WS_2_ is n-type doped. We estimated the field-effect carrier mobility using the equation *μ*=[d*I*_ds_/d*V*_bg_] × [*L*/(*W* × *V*_ds_*C*_i_)], where d*I*_ds_/d*V*_bg_ is the transconductance, *L*, *W* and *C*_i_ are the channel length, channel width and the capacitance between the channel and the back gate per unit area, respectively. The evaluated carrier mobility is 1.7 cm^2 ^V^−1 ^s^−1^, and the ON/OFF current ratio is ∼10^7^. It is worth pointing out that most of the BG-FETs we measured show the mobilities ranging from 1 to 2 cm^2^V^−1^s^−1^ and ON/OFF ratios ranging from 4 × 10^6^ to 5 × 10^7^ (Fig. 4d). These mobilities are ∼100 times larger than those of BG-FETs made by CVD-grown monolayer WS_2_ on SiO_2_/Si substrates (∼0.01 cm^2 ^V^−1 ^s^−1^; ref. 7), and are comparable to those reported for BG-FETs fabricated with mechanically exfoliated WS_2_ (∼10 cm^2 ^V^−1 ^s^−1^; ref. 24). Moreover, the ON/OFF ratios are one to two orders of magnitude larger than those reported so far for monolayer WS_2_ including mechanically exfoliated samples^7^,^24^,^25^. These results give further strong evidence for the high quality of our samples and confirm the great potential of our samples for applications in transistors and integrated circuits.

### Large-area monolayer WS_2_ films and flexible electronics

**Figure 5:**
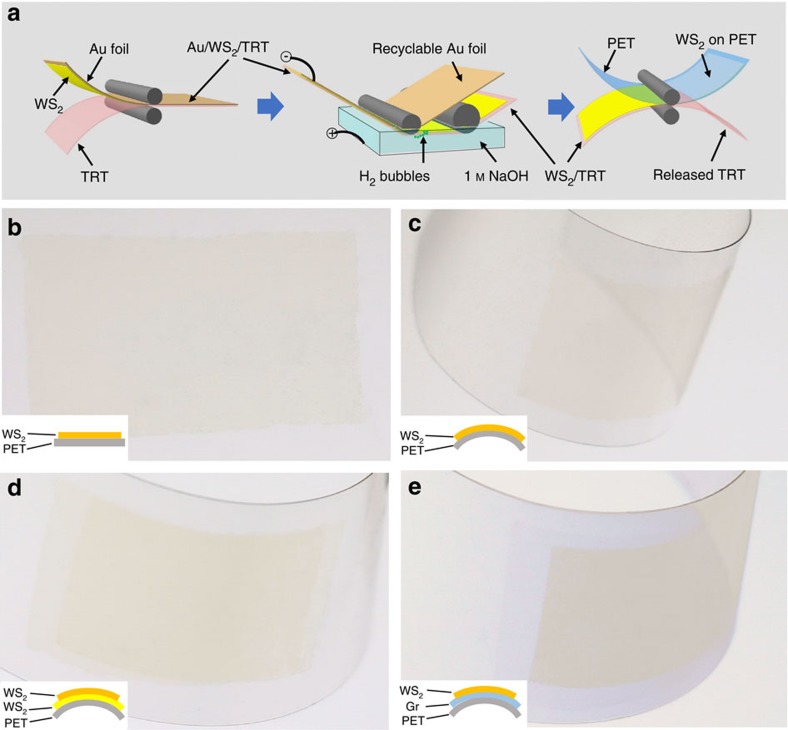
Roll-to-roll/bubbling production of large-area flexible monolayer films and their heterostructures with graphene films. (**a**) Schematic of the roll-to-roll/bubbling process. (**b**–**e**) Photographs of ∼2-inch flexible transparent films of monolayer WS_2_ (**b**,**c**), double-layer WS_2_ (**d**) and graphene/monolayer WS_2_ van der Waals heterostructure (**e**) on PET fabricated by the roll-to-roll/bubbling method. The insets in **b**–**e** show the schematic models of the transferred films. ‘Gr' represents graphene.

Another very important advantage of our method is that the CVD growth and electrochemical bubbling transfer presented here can be integrated in a roll-to-roll technique to produce large-area monolayer WS_2_ films on flexible substrates such as PET and polyethylene naphthalate (PEN) at low cost because of the good flexibility and chemical stability of Au foils. Figure 5a shows a schematic of the roll-to-roll/bubbling production of monolayer WS_2_ films on PET. First, a monolayer WS_2_ film, grown on a flexible Au foil, was attached to a thermal release tape (TRT) by passing between two rollers. Second, the TRT/WS_2_/Au thus obtained was gradually rolled into a 1-M NaOH aqueous solution under a constant current. During this process, the TRT attached to the WS_2_ film was separated from the Au foil by hydrogen bubbles generated by water electrolysis. Third, the monolayer WS_2_ film was detached from the TRT and was attached to a PET film by thermal treatment when the TRT/WS_2_/PET passed between two rollers. It is worth noting that the Au foil was not destroyed during this roll-to-roll bubbling process and can be reused for monolayer WS_2_ growth, which greatly reduces the monolayer WS_2_ production cost. This is different from the traditional roll-to-roll process for graphene transfer, where Cu foils are etched away^28^.

Figure 5b,c shows an ∼2-inch monolayer WS_2_ film on PET fabricated by our roll-to-roll/bubbling method. It shows no visible cracks and similar Raman features to single-crystal monolayer WS_2_ (Supplementary Fig. 25), indicating its intact transfer and high quality. Moreover, this method can be used to produce other 2D materials such as graphene, few-layer WS_2_ films and van der Waals heterostructures of monolayer WS_2_ assembled with other 2D materials. For example, we have fabricated large-area high-quality transparent and flexible double-layer WS_2_ films (Fig. 5d, Supplementary Fig. 25 and Supplementary Fig. 26) and graphene/monolayer WS_2_ heterostructures (Fig. 5e) through layer-by-layer transfer. The roll-to-roll/bubbling production of such 2D materials opens up the possibility for the large-scale use of monolayer WS_2_ in integrated circuits, vertical FETs and photovoltaic devices for transparent flexible electronics and optoelectronics.

**Figure 6:**
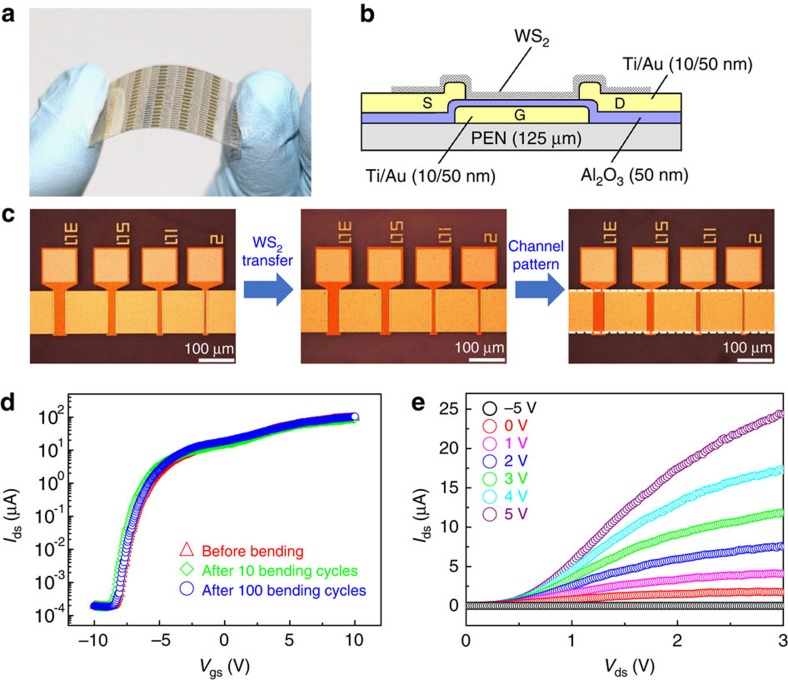
Fabrication and electrical properties of large-area flexible monolayer WS_2_-based FET arrays. (**a**) Photograph of large-area flexible monolayer WS_2_ FET arrays fabricated on a PEN substrate. (**b**) Schematic cross-section of a WS_2_ buried-gate FET. (**c**) Optical images showing the fabrication of monolayer WS_2_ FET arrays. Left panel, pre-patterned electrodes on a PEN substrate. Middle panel, an ∼2-inch monolayer WS_2_ film transferred from a Au foil on the substrate in the left panel using the roll-to-roll/bubbling method. Right panel, the patterned monolayer WS_2_ film (indicated by dotted white lines) to form FET channels. (**d**) Typical logarithmic transfer characteristics of a buried-gate FET showing *μ*=0.99 cm^2 ^V^−1 ^s^−1^ with an ON/OFF ratio of ∼6 × 10^5^. There is no electrical performance degradation after repeated bending to a radius of 15 mm for 100 times. (**e**) Output characteristics of the same device measured at room temperature.

As an example, we fabricated large-area flexible monolayer WS_2_ FET device arrays with buried gates on a PEN substrate (Fig. 6). We used the roll-to-roll/bubbling method described above to transfer an ∼2-inch monolayer WS_2_ film onto the PEN substrate with pre-patterned electrodes made by standard photolithography (Fig. 6c, also see Methods). Note that the photolithography process and roll-to-roll/bubbling method are compatible with the batch fabrication of large-area flexible devices. Figure 6d presents the transfer characteristics of a WS_2_ FET on the flexible PEN substrate, showing n-type characteristics. The mobility and ON/OFF ratio were evaluated to be 0.99 cm^2 ^V^−1 ^s^−1^ and ∼6 × 10^5^, respectively, which are comparable to those of the devices on SiO_2_/Si substrates. Moreover, no electrical performance degradation was observed even after repeated bending to a radius of 15 mm for 100 times. These results indicate that our growth and transfer methods have a great potential for the batch fabrication of large-area WS_2_-flexible devices. The output characteristics of the same devices show a saturation behaviour at a high source-drain bias (Fig. 6e); however, a nonlinear current at a low source-drain bias indicates a non-ideal ohmic contact between Au electrodes and WS_2_ channel. We believe that the electrical performance including the carrier mobility and ON/OFF ratio of the flexible WS_2_ FET presented here can be further improved if given a careful selection of electrode metals with different work functions to decrease the Schottky barrier height at WS_2_/metal interface.

## Discussion

In summary, we report the uniform growth of high-quality monolayer WS_2_ single crystals of the order of millimetres and large-area continuous films by ambient-pressure CVD using Au foils as the growth substrate. Similar to the CVD growth of graphene on copper, the catalytic activity of gold together with its low solubility for tungsten allow self-limited catalytic growth of a uniform monolayer of WS_2_ on its surface. Furthermore, the weak interaction between monolayer WS_2_ and gold enables the intact transfer of the WS_2_ onto arbitrary substrates without sacrificing the Au foil by using the electrochemical bubbling method. The large WS_2_ single crystals show high crystal quality and excellent optical and electrical properties comparable or superior to mechanically exfoliated samples. We also demonstrate that this CVD growth and electrochemical bubbling transfer can be integrated in a roll-to-roll technique for the production of large-area transparent and flexible monolayer WS_2_ films and their heterostructures with graphene films, and batch fabrication of large-area-flexible monolayer WS_2_ film transistor arrays with electrical performance comparable to those on SiO_2_/Si substrates. These results open up the possibility of using monolayer WS_2_ in next-generation electronics, optoelectronics and valleytronics.

## Methods

### Ambient-pressure CVD growth of uniform monolayer WS_2_ on Au foils

A piece of Au foil (99.95 wt%, 25–100-μm thick) was finely polished and annealed at 1,040 °C for over 10 h before the first use, and the polished face was placed face-down above a small quartz boat containing 200 mg WO_3_ powder (99.998 wt%) in the heating zone of a 1-inch horizontal tube furnace. Another small quartz boat containing 100 mg S powder (99.9995, wt%) was placed upstream outside the furnace, where the temperature was independently controlled (Supplementary Fig. 1). The Au foil was heated to 800 °C in an Ar atmosphere (200 s.c.c.m.) and annealed for 10 min to remove organics adsorbed on its surface. The S powder was then heated to 200–240 °C to generate S vapour to initiate the growth of WS_2_. After CVD growth for a certain time (4 h for a millimetre-size single-crystal WS_2_ sample), the furnace was cooled to room temperature naturally. The Ar gas flow rate remained constant (10–500 s.c.c.m.) during the growth and cooling processes.

### Bubbling transfer of monolayer WS_2_ from Au foils

Similar to the transfer of graphene grown on Pt (ref. 45), the Au foil with monolayer WS_2_ was first spin-coated with 550-k PMMA (4 wt% dissolved in ethyl lactate) at 3,000 r.p.m. for 1 min followed by curing at 180 °C for 5 min. Then, the PMMA/monolayer WS_2_/Au was used as the cathode in a 1-M NaOH solution with a Pt foil as the anode. After applying a constant current of 50 mA for 30 s, the PMMA/monolayer WS_2_ layer was separated from the Au foil by H_2_ bubbles generated by water electrolysis. After that, the PMMA/monolayer WS_2_ was immersed in deionized water and collected by a target substrate. Finally, the PMMA layer was removed by warm acetone (50 °C), rinsed with isopropanol and dried by a N_2_ flow. For TEM samples, the PMMA layer was dissolved with acetone droplets and dried naturally.

### Structure and optical property characterization

The morphology of the monolayer WS_2_ and Au foils was characterized using an optical microscope (Nikon Eclipse LV100) and scanning electron microscopy (SEM; Nova NanoSEM 430, 15 kV), and the thickness was measured using AFM (Veeco Dimension 3100, tapping mode). X-ray photoelectron spectroscopy (ESCALAB 250) was used to characterize the chemical composition of the Au foils before and after different treatments. HRTEM imaging was performed on a field emission TEM (FEI Tecnai F20, 200 kV); STEM imaging was performed on an aberration-corrected and monochromated TEM (FEI Titan^3^ G2 60–300 S/TEM) operating at 60 kV; and SAED measurements were performed on a TEM operating at 120 kV (FEI Tecnai T12). Raman and PL spectra/maps were collected with a confocal Raman spectrometer (Jobin Yvon LabRAM HR800) using a 532-nm laser as the excitation source. The laser spot size was ∼1 μm and the laser power on the sample surface was kept below 60 μW to avoid any heating effects. Ultraviolet-visible absorption spectra were collected with a Ultraviolet-vis NIR spectrometer (Varian Cary 5000).

### Electrical property measurements of monolayer WS_2_ on SiO_2_/Si

BG-FETs were fabricated on n-doped Si substrates with 290-nm-thick SiO_2_ as the dielectric layer. The monolayer single-crystal WS_2_ domains were patterned through exposure to electron-beam lithography followed by reactive ion etching (O_2_ plasma treatment for 50 s at 100 W). Ti/Au (5/30 nm) was used as the source and drain electrodes. All the BG-FETs were directly measured after fabrication, without annealing, with a semiconductor analyser (Keithley 4200) inside a probe station (Lakeshore TTP-4) in vacuum at room temperature.

### Fabrication and electrical property measurements of large-area flexible monolayer WS_2_ FET arrays

First, the buried-gate electrodes (Ti/Au: 10/50 nm) were fabricated on PEN substrates (Teijin DuPont Films; thickness, 125 μm) by standard photolithography, electron-beam evaporation and lift-off processes. A 50-nm Al_2_O_3_ insulator layer was then deposited by an atomic layer deposition technique using trimethylaluminium and H_2_O at 145 °C. Contact windows for the gate electrodes were opened using photolithography and reactive ion etching. The source and drain electrodes were fabricated by the similar processes to the gate electrodes. Second, we used the roll-to-roll/bubbling method to transfer an ∼2-inch monolayer WS_2_ film onto the substrate with patterned electrodes. Third, the WS_2_ film outside the channel area was removed by photolithography and O_2_ plasma treatment for 2 min at 200 W. The electrical property measurements of these flexible devices were same as those for the devices on SiO_2_/Si substrates shown above.

### DFT calculations on the sulphurization of WO_3_ with and without the presence of Au substrate

The first-principles calculations were performed using the Vienna *Ab initio* Simulation Package^50^,^51^,^52^,^53^ with the projector augmented wave method^54^,^55^. The Perdew–Burke–Ernzerhof functional^56^ for the exchange-correlation term was used for all calculations. The projector augmented wave method was used at a plane-wave cutoff of 400 eV to describe the electron–ion interaction. The W_3_O_9_ cluster was used to represent the WO_3_ species for WS_2_ growth. The Au(111) surface was modelled by periodically repeated slab consisting of three Au atom layers and the vacuum layer with a thickness of 15 Å. Only the Gamma point was used because of the large supercell of the Au(111) surface, and the Au atoms in the bottom layer were fixed for all calculations, whereas the rest of the atoms were allowed to fully relax during geometry optimization with the convergence criteria of 1 × 10^−6 ^eV in total free energy. To achieve the minimum energy paths for S_2_ dissociation, S atom diffusion on the Au(111) surface, and the possibly initial step for the reduction of W_3_O_9_ cluster by S_2_ molecule at sulphur atmosphere and by S atoms on the Au(111) surface, we employed the climbing image nudged elastic band method^57^, and the convergence tolerance of force on each atom for searching minimum energy path was set to be 0.05 eV Å^−1^.

## Additional information

**How to cite this article:** Gao, Y. *et al*. Large-area synthesis of high-quality and uniform monolayer WS_2_ on reusable Au foils. *Nat. Commun.* 6:8569 doi: 10.1038/ncomms9569 (2015).

## Supplementary Material

Supplementary Information Supplementary Figures 1-26, Supplementary Note 1 and Supplementary References.
