# Supplementary material for: Large-area synthesis of high-quality and uniform monolayer WS2 on reusable Au foils
Source: Nat Commun. 2015 Oct 9;6:8569. doi: 10.1038/ncomms9569 (PMC4633959; doi:10.1038/ncomms9569)
Supplement: Supplementary Information — Supplementary Figures 1-26, Supplementary Note 1 and Supplementary References. [file ncomms9569-s1.pdf]

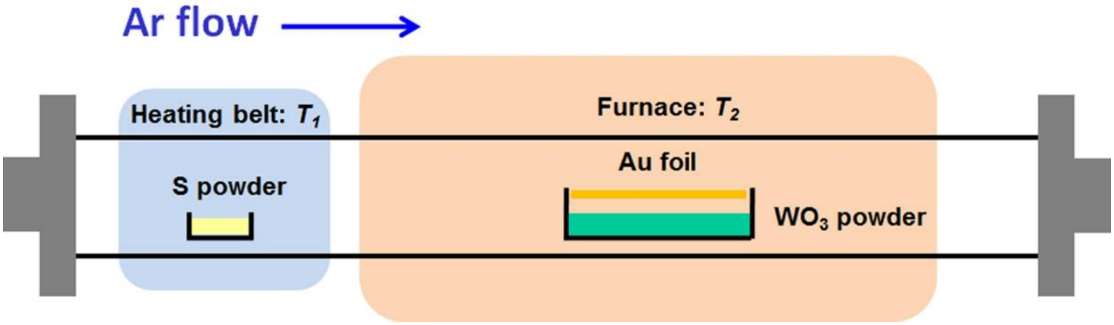

Supplementary Figure 1. Schematic for the growth of high-quality uniform monolayer WS_2 by ambient-pressure CVD.

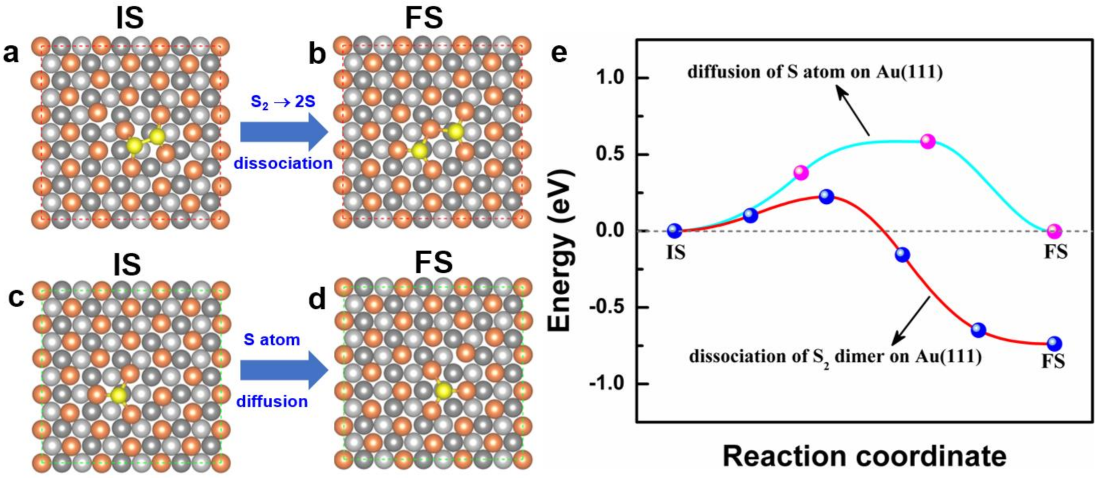

Supplementary Figure 2. Schematic structures of the initial state (IS) and the final state (FS) for **(a)** → **(b)** S_2 dissociates into two S atoms, **(c)** → **(d)** S atom diffusion on Au(111) surface, and **(e)** the calculated minimum energy paths for these two processes. The Au atoms in the bottom, the middle, and the top layers are represented by dark grey, light grey, and brown balls, respectively. The S atoms are represented by the yellow balls. It can be found that, on Au(111) surface, the S_2 dimer can be easily dissociated into S atoms and the obtained S atoms also can diffuse very easily because of the very low barriers for these two processes (0.22 eV for S_2 dissociation, and 0.58 eV for S atom diffusion).

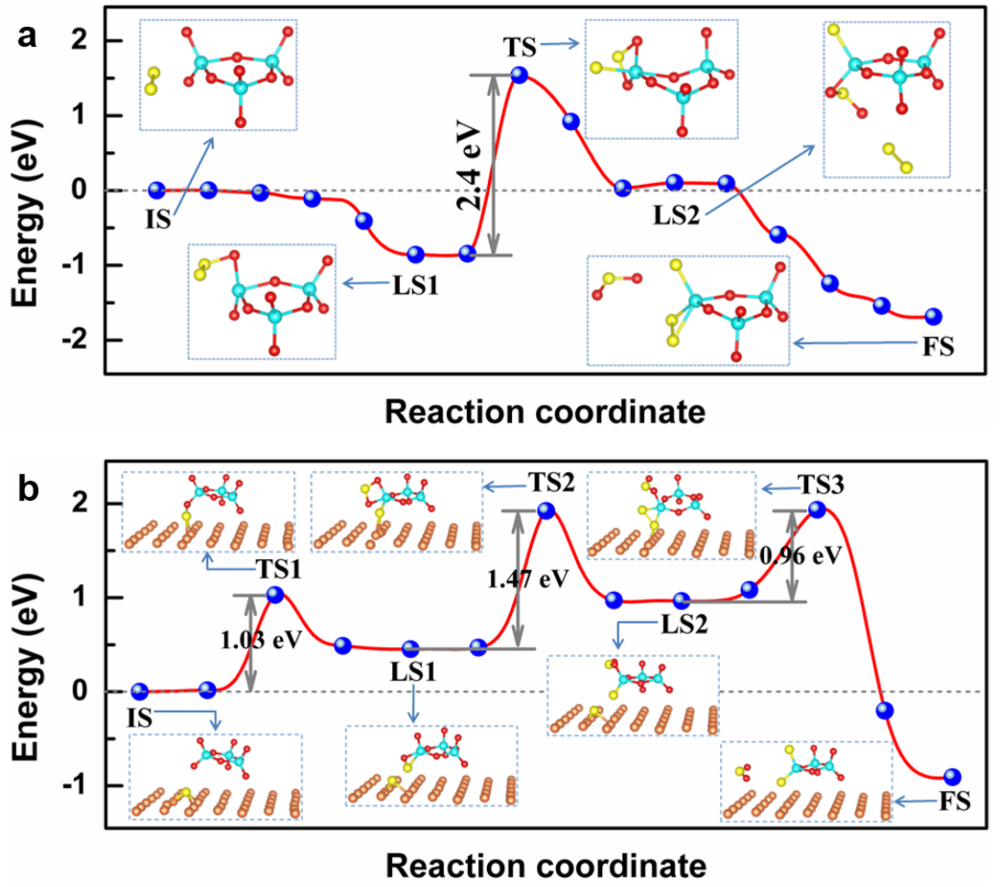

Supplementary Figure 3. The calculated minimum energy paths for the possible first step of W_3O_9 reduction by **(a)** S_2 molecules in sulphur atmosphere and **(b)** S atoms on Au(111) surface. Schematic structures of the corresponding initial states (IS), local states (LS) and final state (FS) are also presented. The O, S, W, and Au atoms are represented by the red, yellow, cyan, and brown balls, respectively. Only the topmost atomic layer of Au(111) surface is shown for simplicity. The second S_2 molecule is introduced from LS2 in **a**, and the second and the third S atoms are introduced from LS1 and LS2 in **b**, respectively. It can be found that S atoms on Au(111) can reduce W_3O_9 cluster into $W_3O_7S_2$ with a moderate energy barrier of 1.47 eV, which is much lower than that (2.39 eV) of direct sulfurization of W_3O_9 cluster by S_2 dimer in sulphur atmosphere without the presence of Au substrate.

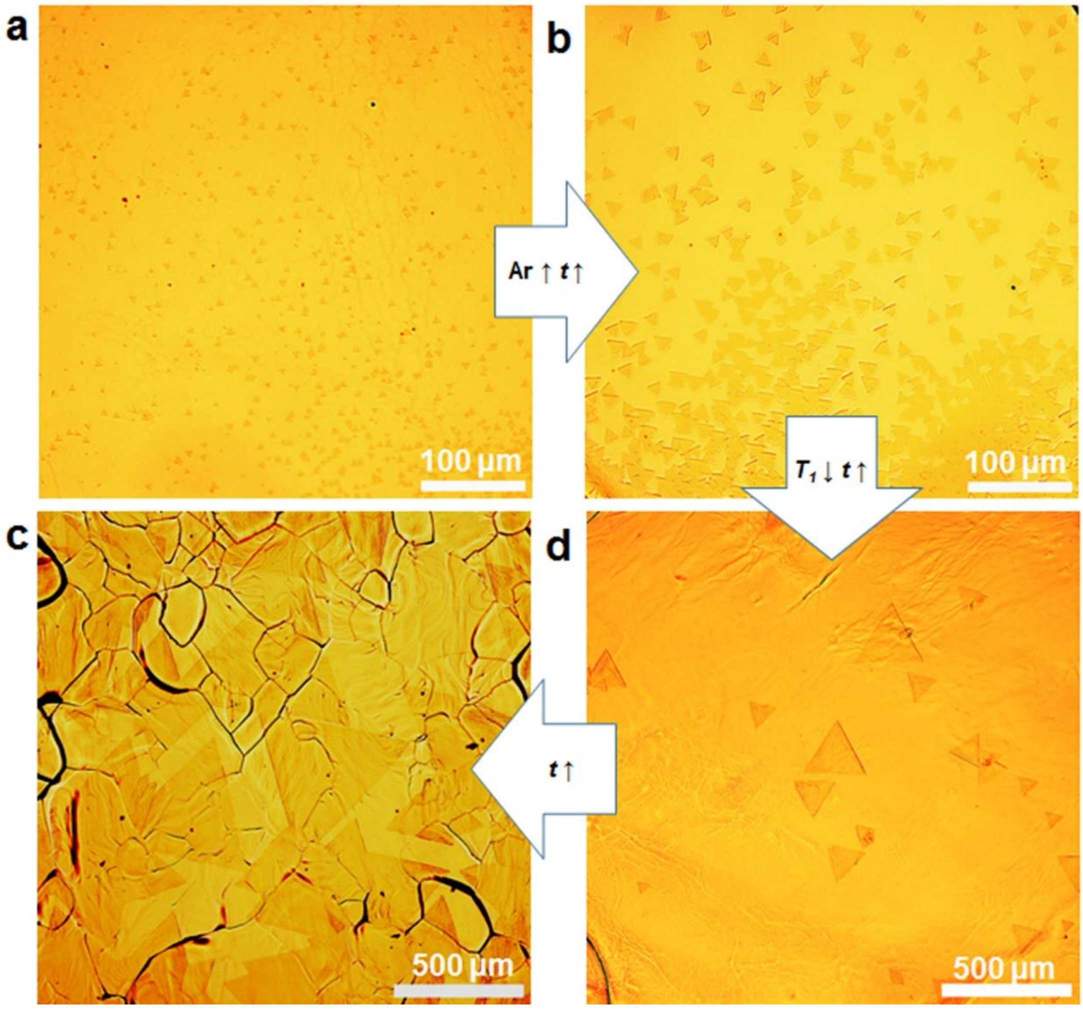

Supplementary Figure 4. Monolayer single-crystal WS_2 domains prepared at different conditions: (**a**) T_1 = 240 °C, Ar = 10 sccm, t = 10 min; (**b**) T_1 = 240 °C, Ar = 200 sccm, t = 0.5 h; (**c**) T_1 = 200 °C, Ar = 200 sccm, t = 4 h; (**d**) T_1 = 200 °C, Ar = 200 sccm, t = 1 h. T_1 refers to the heating temperature of the sulphur powder, and t refers to the growth time. All the other experimental parameters are the same.

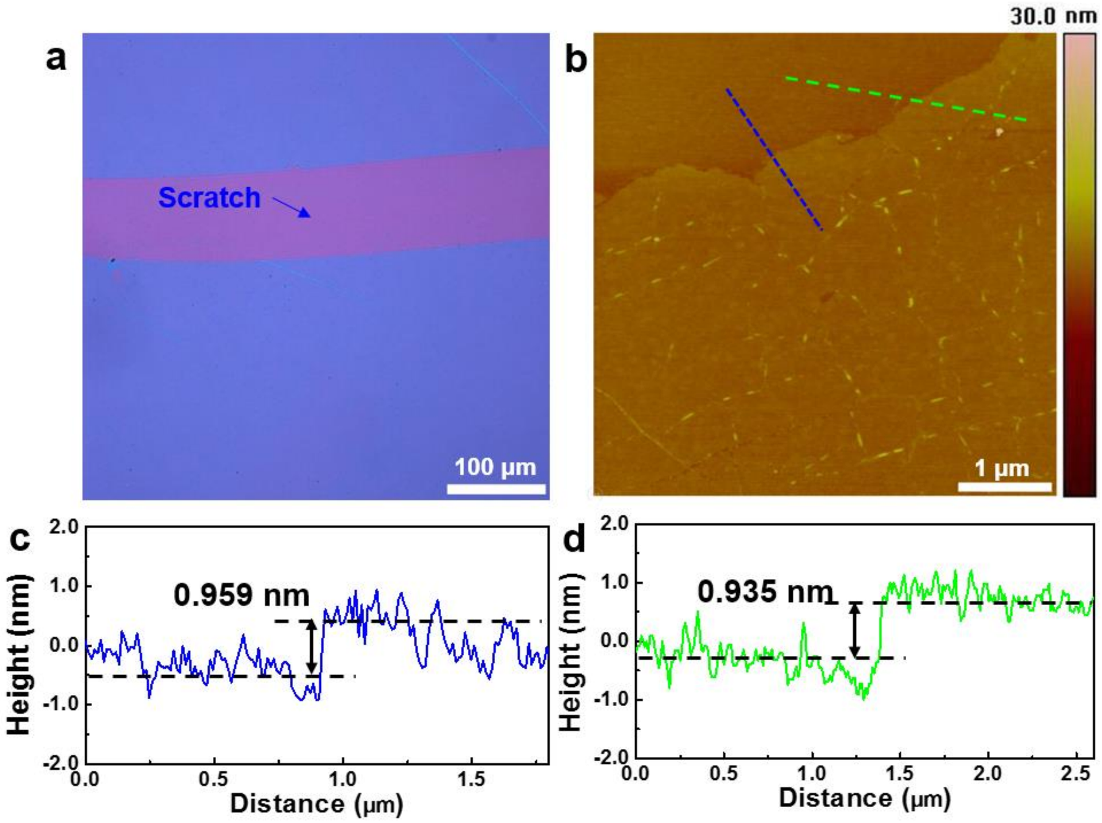

Supplementary Figure 5. (**a**) Optical image of the WS_2 sample grown on a Au foil and then transferred onto a SiO_2/Si substrate, in which a scratch was made with a plastic sheet for AFM measurements. (**b**) Typical AFM image of the scratch edge in WS_2 sample in **a**. The yellow lines in the WS_2 sample represent the wrinkles, which were formed probably due to the thermal expansion difference between WS_2 and Au. (**c**), (**d**) Height profiles across the scratch edge in **b**.

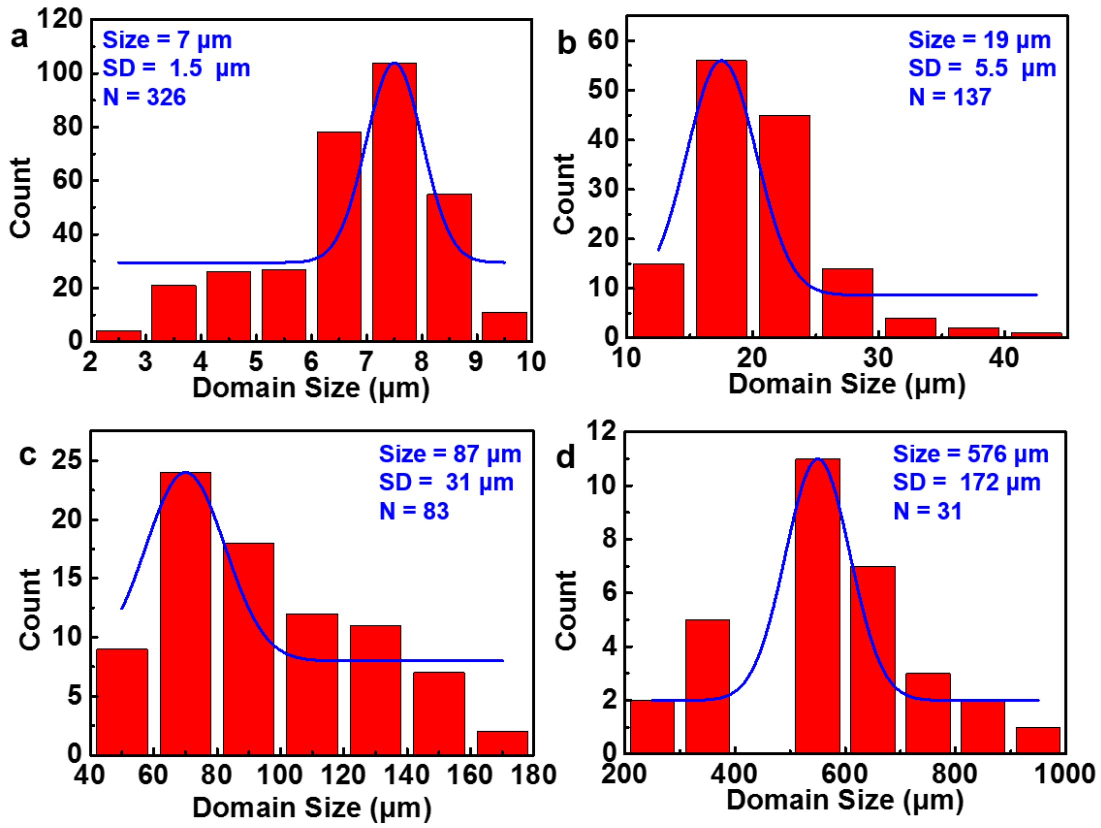

Supplementary Figure 6. Size distribution of monolayer WS_2 single-crystal domains prepared at different conditions. **(a)** T_1 = 240 ºC, Ar = 10 sccm, t = 10 min; **(b)** T_1 = 240 ºC, Ar = 200 sccm, t = 45 min **(c)** T_1 = 220 ºC, Ar = 200 sccm, t = 1h; **(d)** T_1 = 200 ºC, Ar = 500 sccm, t = 4h. All other experimental parameters are the same. 'SD' represents standard deviation.

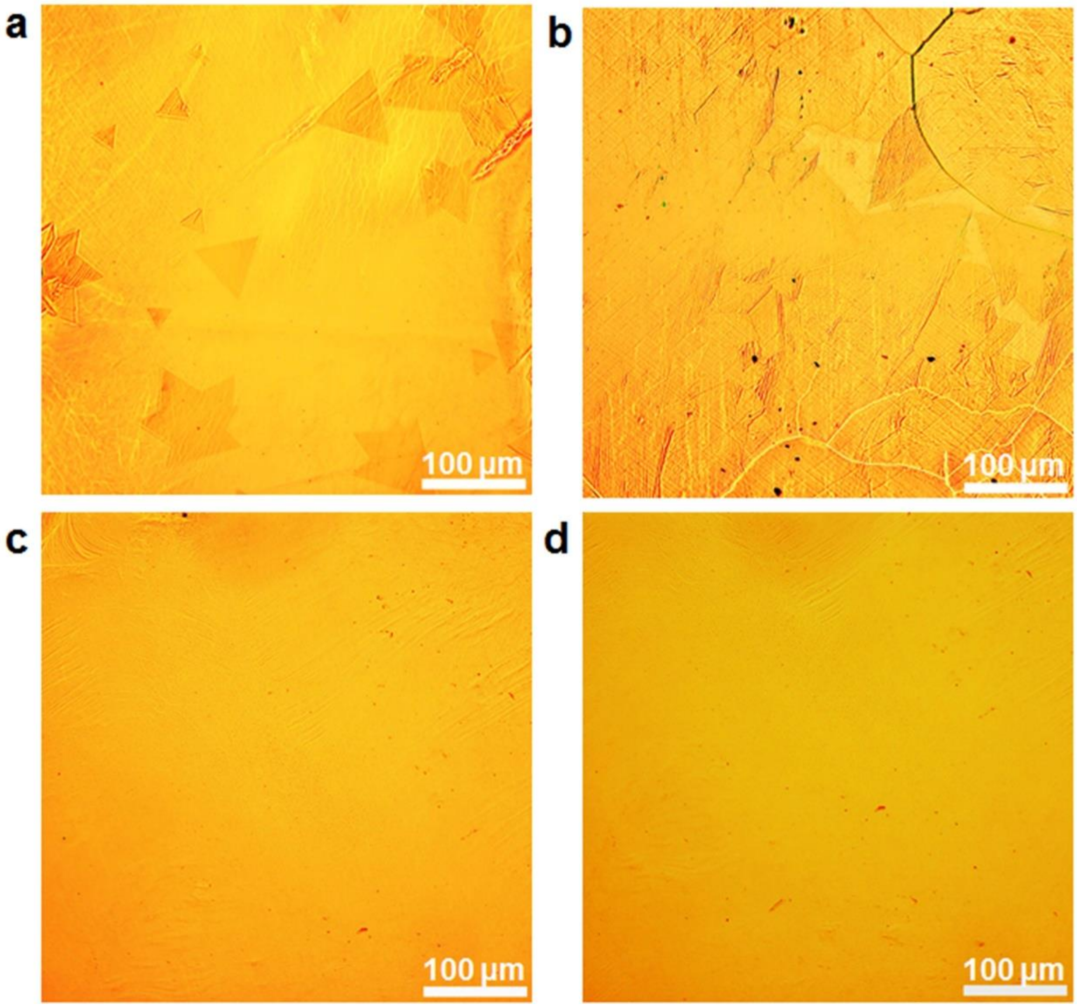

Supplementary Figure 7. Uniform monolayer WS_2 prepared with growth times of (**a**) 0.5 h, (**b**) 1 h, (**c**) 1.5 h, and (**d**) 4 h. Note that, with increasing growth time, adjacent monolayer WS_2 domains (**b**) expanded, joined up, and (**c**) finally formed a continuous film, but no additional layers were then formed on the surface of the film as can be seen by comparing **c** and **d**. T_1 = 220 °C, Ar = 10 sccm.

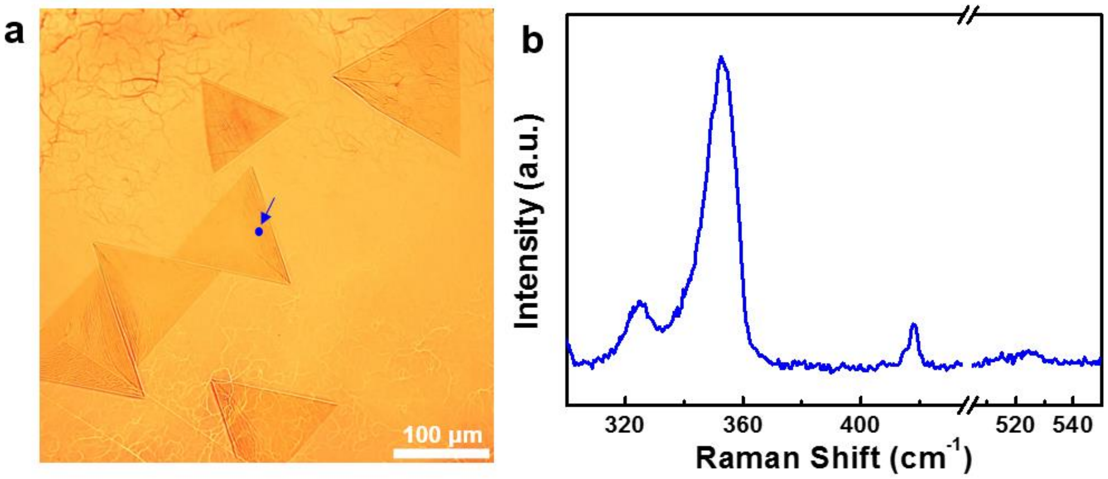

Supplementary Figure 8. (**a**) Optical image of a Au foil loaded after the growth of continuous WS_2 films and then subjected to CVD growth for 60 min. (**b)** Raman spectrum taken at the blue dot position in **a**, indicating the growth of monolayer WS_2.

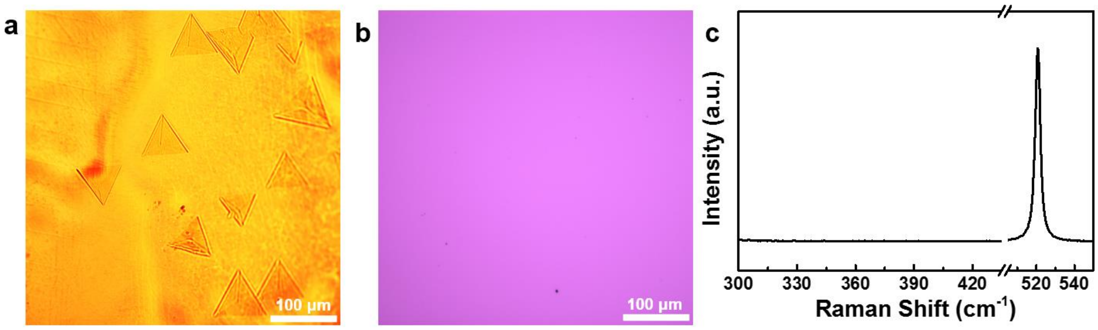

Supplementary Figure 9. Optical images of the (**a**) Au foil and (**b**) SiO_2/Si substrate after WS_2 growth at conditions: T_1 = 220 ºC, Ar = 200 sccm, t = 1h. It can be found that many WS_2 domains are formed on the Au foil. In contrast, Raman measurements (**c**) show that there is nothing formed on SiO_2/Si substrate.

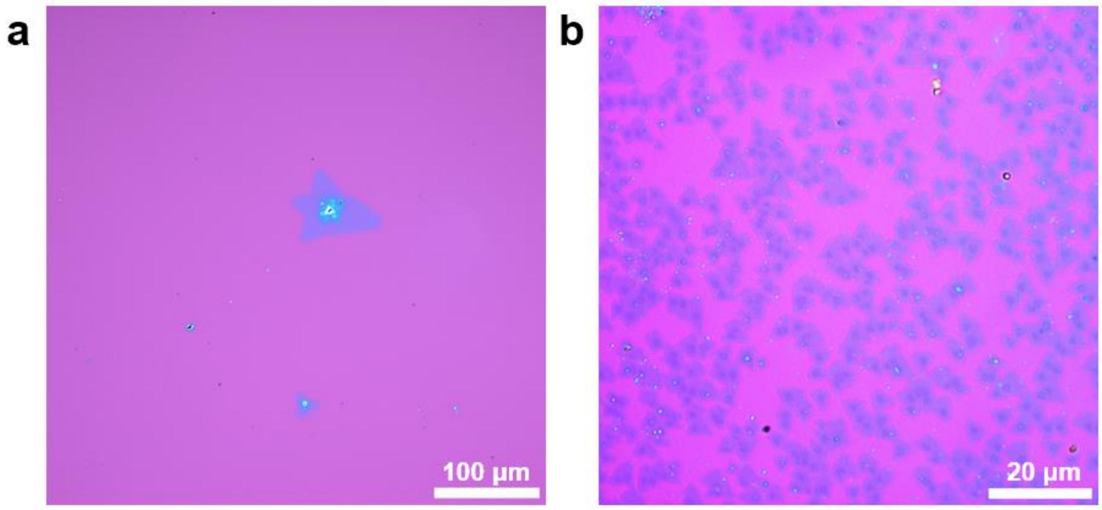

Supplementary Figure 10. Optical images of the WS_2 domains **(a)** grown on SiO_2/Si substrate and **(b)** grown on Au foil and then transferred onto SiO_2/Si substrate. Growth conditions: T_1 = 250 ºC, Ar = 200 sccm, t = 1 h, and the evaporation area of S powder was increased by ~10 times compared to that used for WS_2 growth shown in Supplementary Fig. 9. It can be found that, under the same conditions, a few WS_2 domains are formed on SiO_2/Si substrate, while many multilayer WS_2 domains are formed on Au foil.

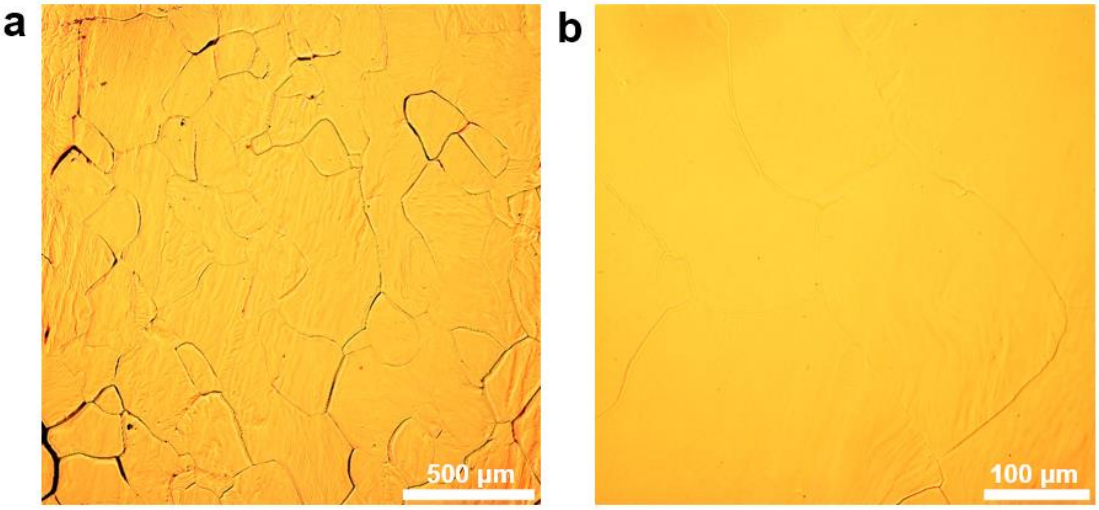

Supplementary Figure 11. (a) Optical image of a Au foil after a two-step growth: Au foil first reacts with WO_3 without the presence of sulphur, and then reacts with sulphur without the presence of WO_3. **(b)** Zoom-in image of **a**, showing no WS_2 formed. To completely remove the residual WO_3 in the CVD system after the reaction of Au substrate with WO_3, we changed a new quartz tube and a new quartz boat to hold the obtained Au substrate, and then continued the reaction with sulphur without the presence of WO_3.

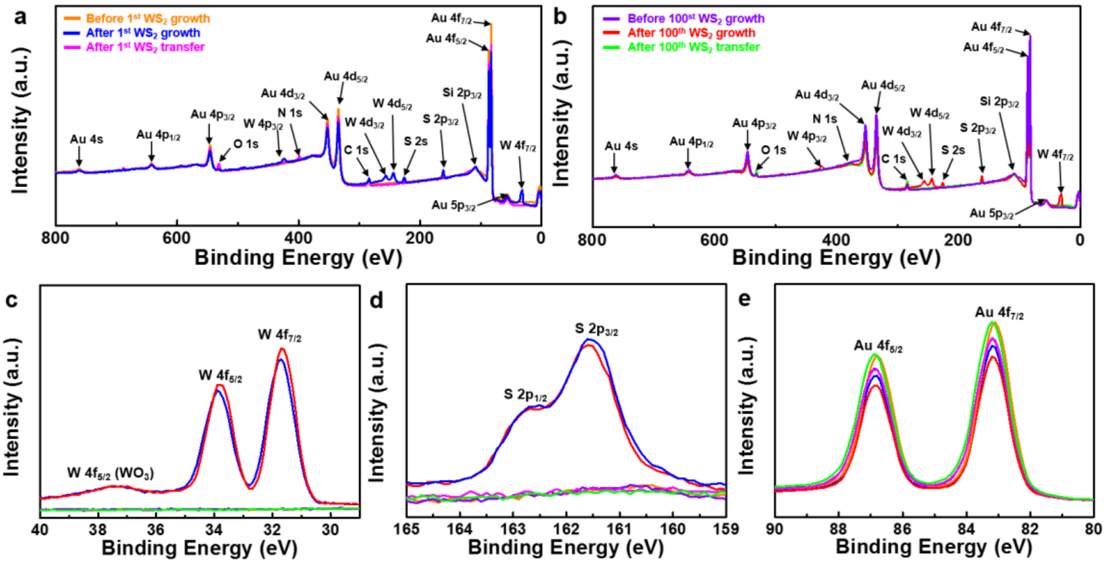

Supplementary Figure 12. XPS spectra of the Au substrate surface (**a**) before the 1st WS_2 growth, after the 1st WS_2 growth, and after bubbling transfer following the 1st WS_2 growth; (**b**) before the 100th WS_2 growth, after the 100th WS_2 growth, and after bubbling transfer following the 100th WS_2 growth. (**c**) W 4f, (**d**) S 2p, and (**e**) Au 4f XPS spectra for the same Au substrate after different treatments (corresponding to the treatments in **a** and **b** for the same colour curves).

It can be found that the Au foils repeated used for different times (after CVD growth and bubbling transfer) have the same chemical composition with the fresh Au foil. The unchanged chemical composition enables the repeated use of Au foils for uniform growth of monolayer WS_2 with high reproducibility.

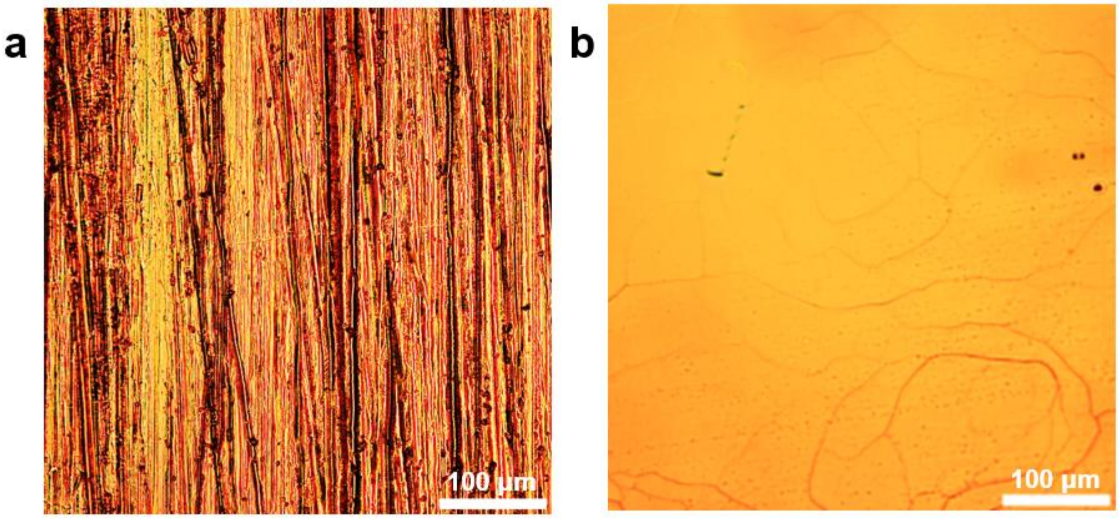

Supplementary Figure 13. Optical images of a Au foil (**a**) without polishing and annealing, and (**b**) after polishing and annealing at 1040 ºC for over 10 h before the 1st CVD growth. Note that the Au surface becomes smooth after polishing and annealing, which is important for the growth of large-size monolayer WS_2 single crystals.

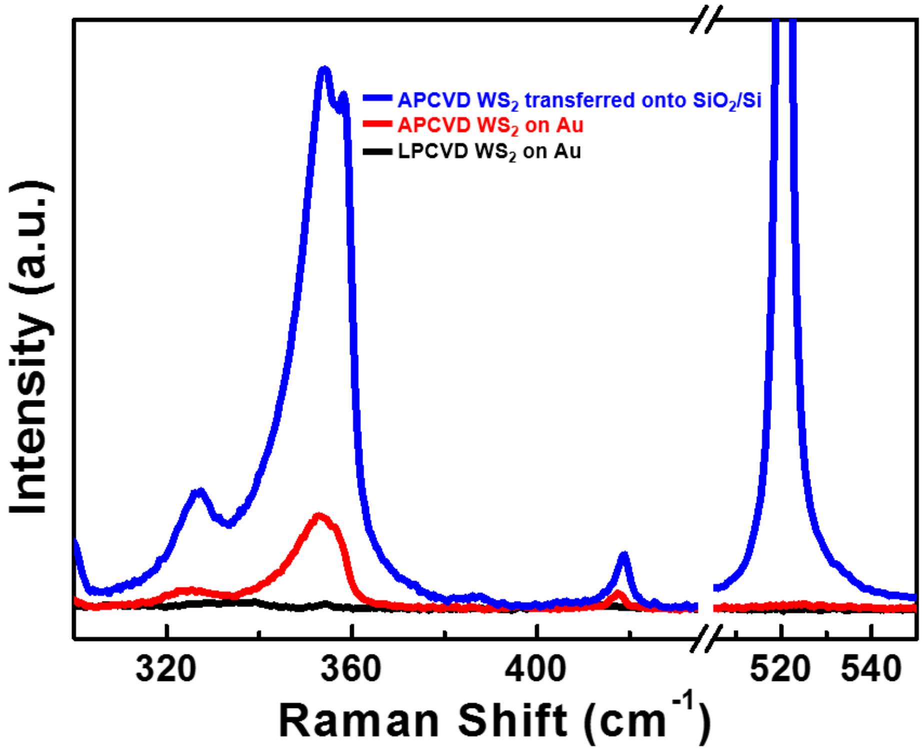

Supplementary Figure 14. Raman spectra of monolayer WS_2 (red plot) grown on a Au foil by ambient-pressure CVD, (blue plot) after transfer onto a SiO_2/Si substrate. The black plot is the spectrum for monolayer WS_2 grown on a Au foil by low-pressure CVD. All spectra were measured with same laser power and accumulation time. Note that Raman peaks are almost invisible in the black line, indicating a strong interface interaction between the WS_2 and Au. In contrast, the WS_2 grown on a Au foil by ambient-pressure CVD shows strong Raman peaks, and its A_{1g} mode peak is downshifted ~1 cm^{-1} compared to that of the WS_2 transferred onto the SiO_2/Si substrate, which indicate a weak interface interaction between the WS_2 and the Au foil.

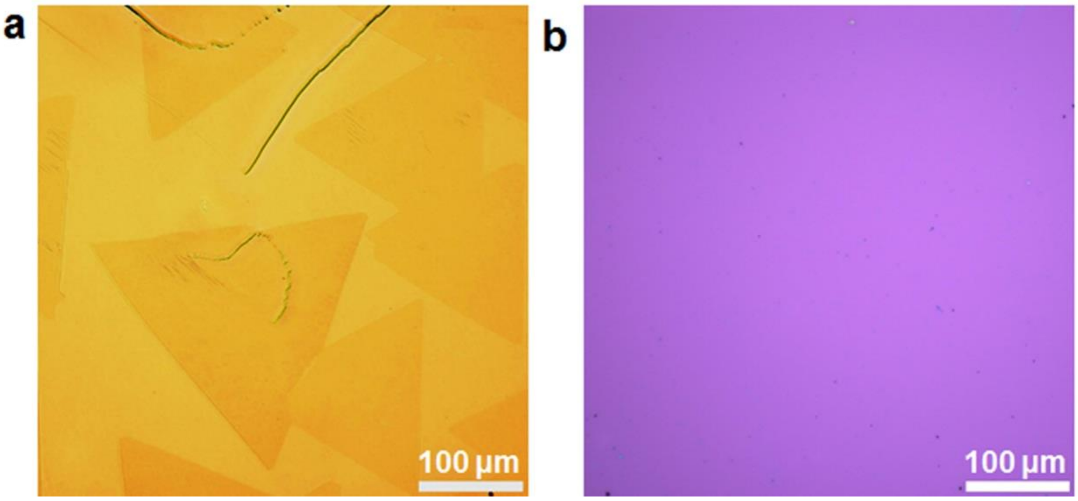

Supplementary Figure 15. (**a**) Optical image of monolayer single-crystal WS_2 domains grown on a Au foil by low-pressure CVD. (**b**) Optical image of a SiO_2/Si substrate after transferring the WS_2 in **a** by the electrochemical bubbling method. There are no WS_2 domains on the SiO_2/Si substrate after transfer, indicating that the WS_2 prepared by low-pressure CVD cannot be transferred by the electrochemical bubbling method because of the strong interface interaction between monolayer WS_2 and the Au foil.

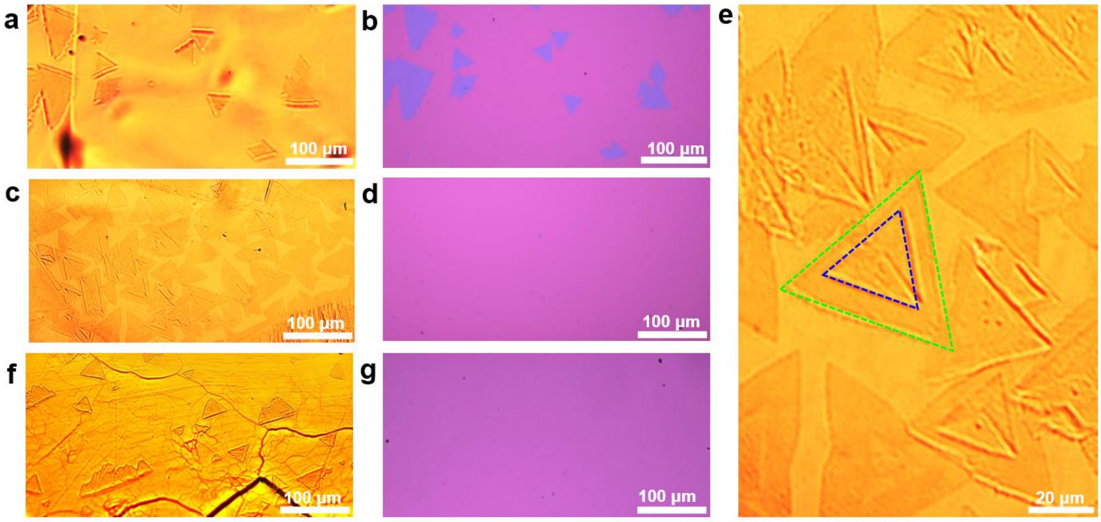

Supplementary Figure 16. (**a**), (**b**) Optical images of monolayer single-crystal WS_2 domains (**a**) grown on a Au foil by ambient-pressure CVD and (**b**) transferred onto SiO_2/Si substrate by the bubbling transfer method. (**c**), (**d**) Optical images of monolayer single-crystal WS_2 domains (**c**) grown on a Au foil by ambient-pressure CVD followed by low-pressure CVD growth (96 Pa, 5 min) and (**d**) transferred onto a SiO_2/Si substrate by the bubbling transfer method. (**e**) Zoom-in image of **c** shows the successive growth of WS_2 domains (the smoother area enclosed between the green dash line and blue dash line) by low-pressure CVD after the formation of original WS_2 domains (the rougher area enclosed by blue dash line) by ambient-pressure CVD. (**f**), (**g**) Optical images of monolayer single-crystal WS_2 domains (**f**) grown on a Au foil by ambient-pressure CVD followed by low-pressure annealing at 800 ºC for 10 min and (**g**) transferred onto a SiO_2/Si substrate by the bubbling transfer method. Note that no WS_2 can be transferred by the bubbling method for the samples grown on a Au foil by ambient-pressure followed by low-pressure CVD growth or annealing.

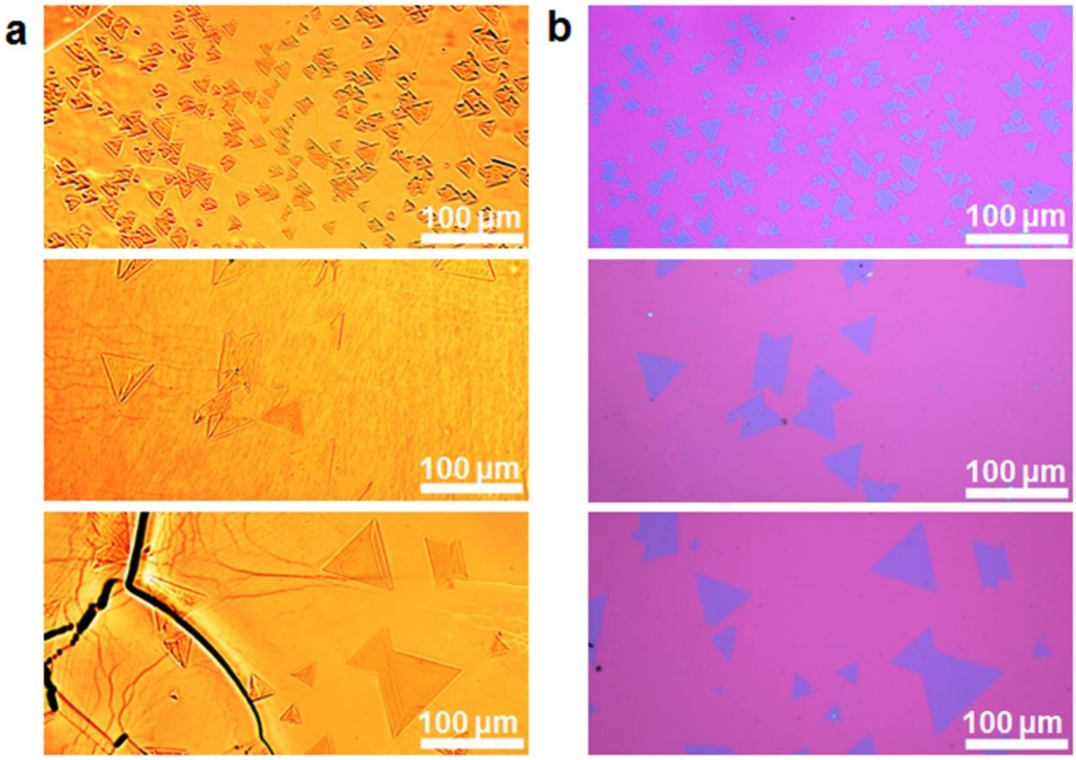

Supplementary Figure 17. (**a**) Optical images of monolayer single-crystal WS_2 domains grown on Au foils by ambient-pressure CVD and (**b**) after transfer onto SiO_2/Si substrates by the electrochemical bubbling method. Note that all the monolayer single-crystal WS_2 domains have been successfully transferred onto the SiO_2/Si substrate without structural damage.

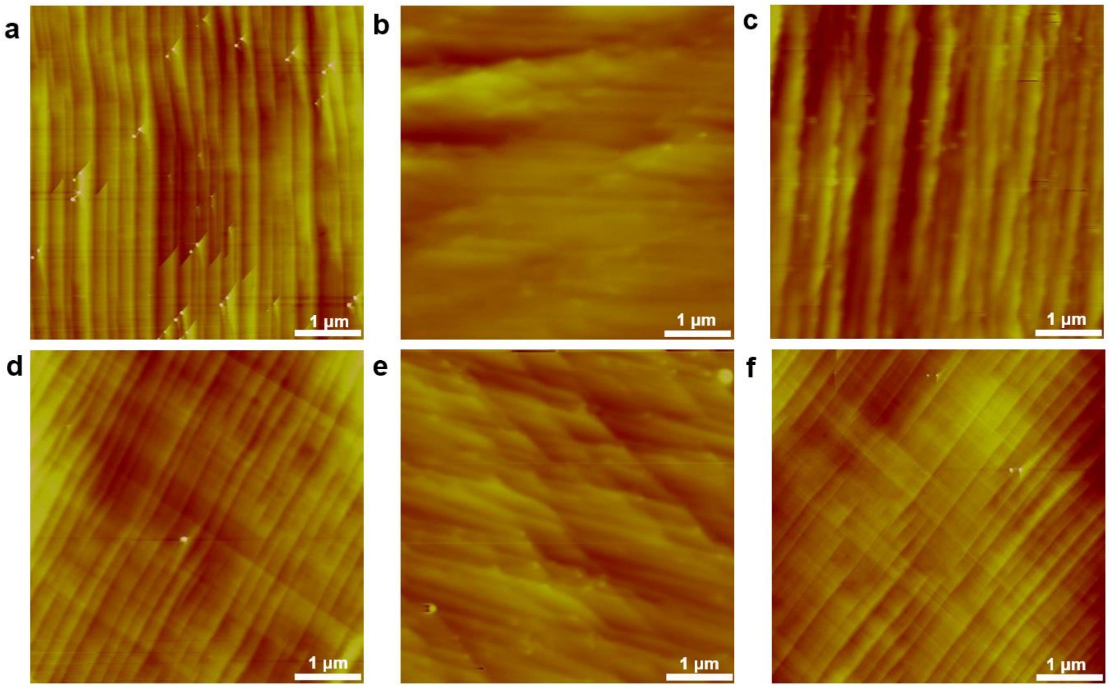

Supplementary Figure 18. AFM images of the Au surface **(a)** after annealing before the 1st WS_2 growth, **(b)** after the 1st WS_2 growth, **(c)** after bubbling transfer following the 1st WS_2 growth, (**d**) after annealing after the 1st WS_2 growth and transfer, **(e)** after the 100th WS_2 growth, **(f)** after annealing after the 100th WS_2 growth and transfer. It can be found that the surface of the Au foil is smooth after annealing before initiating the 1st CVD growth. However, it becomes rough after CVD growth and following bubbling transfer, and the annealing can recover the smooth nature of the surface.

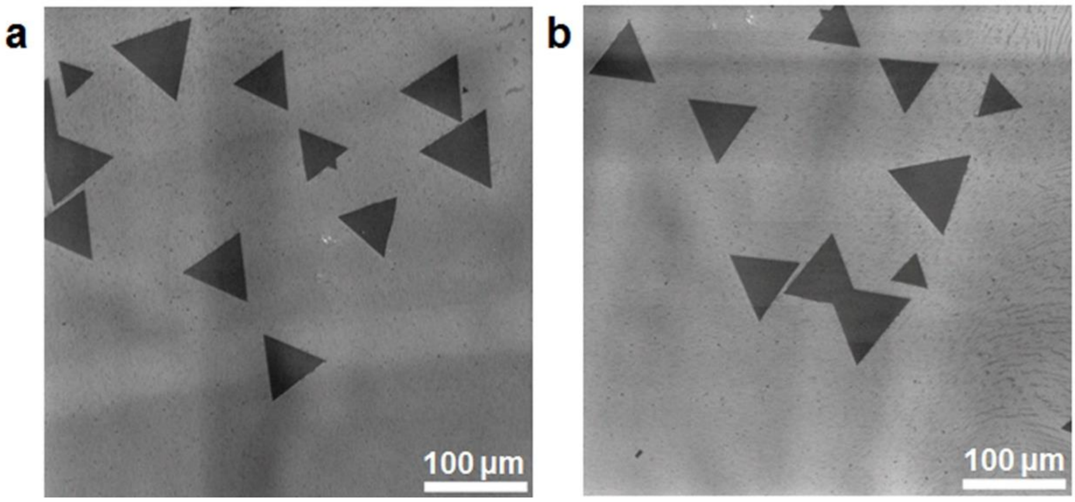

Supplementary Figure 19. SEM images of monolayer single-crystal WS_2 domains grown on the same Au foil after repeated uses by ambient-pressure CVD. (**a**) The first time, and (**b**) after more than 500 times, showing no structural difference.

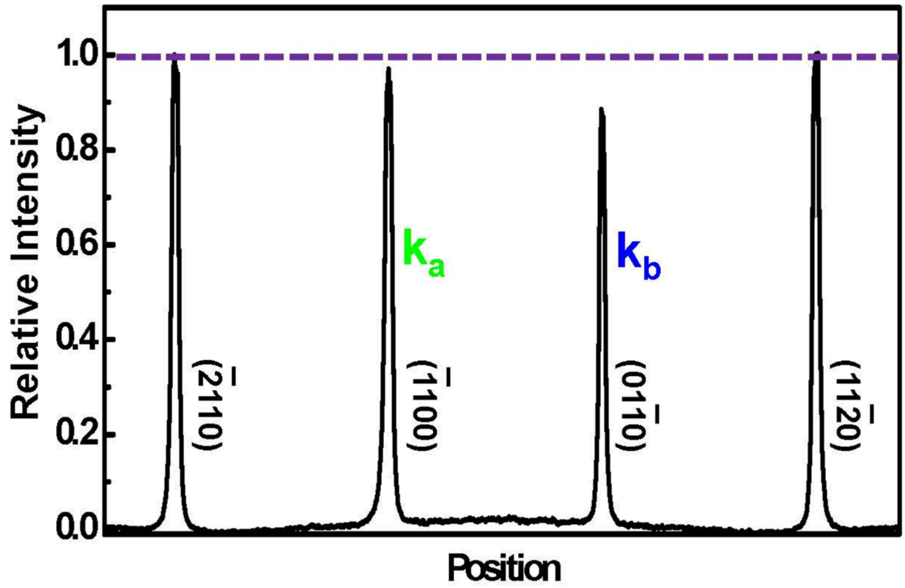

Supplementary Figure 20. Intensity profile of the experimentally measured diffraction spots along the dotted red line in Fig. 2b in the main text, normalized to the height of the $[\bar{2}110]$ peak. The higher intensity $\mathbf{k_a}$ spots are related to the W sublattices.

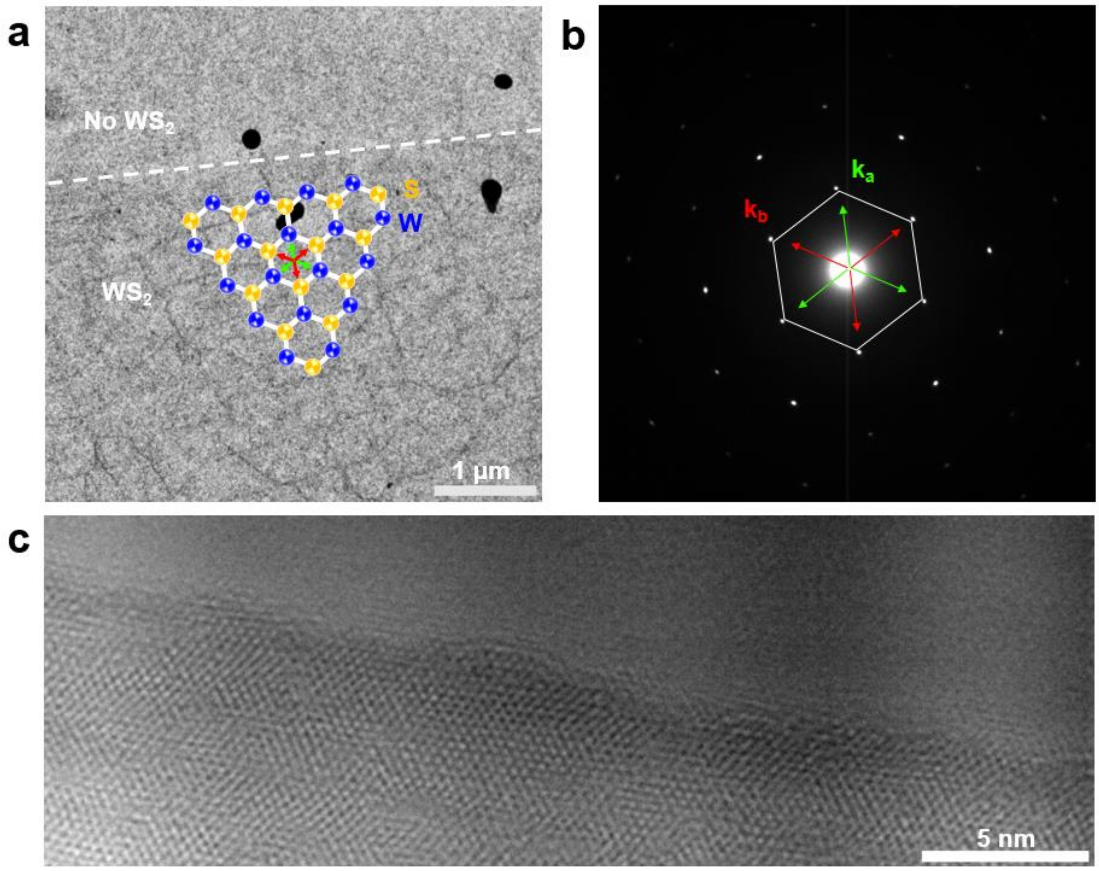

Supplementary Figure 21. (a) Bright-field image of an edge of a monolayer WS_2 domain. **(b)** SAED pattern of the sample in **a**, showing the edge in **a** has zigzag orientation. (c) HRTEM image of the edge in **a**.

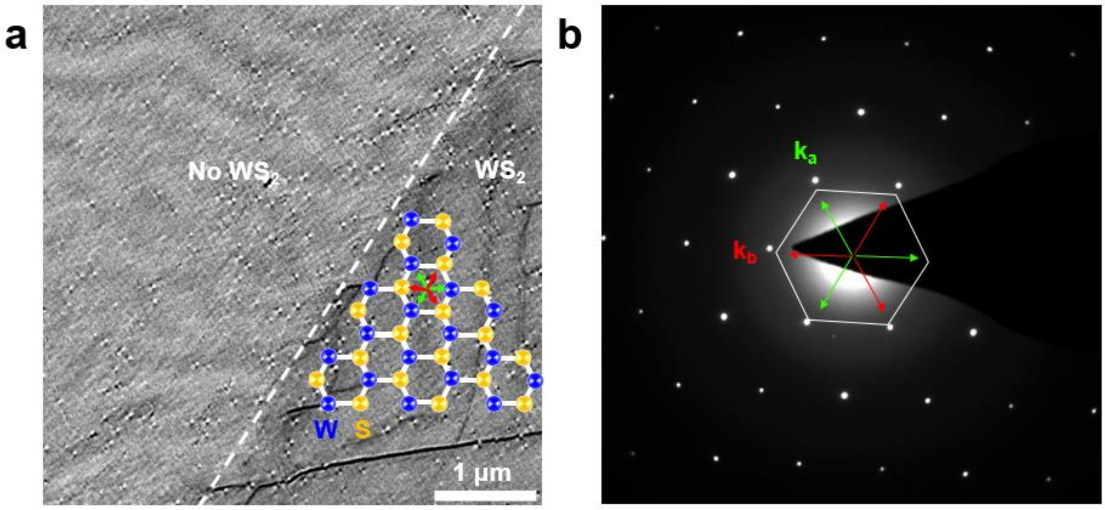

Supplementary Figure 22. (a) Bright-field image of an edge of the monolayer WS_2 domain in Fig. 2a. **(b)** The corresponding SAED pattern, showing the edge has armchair orientation.

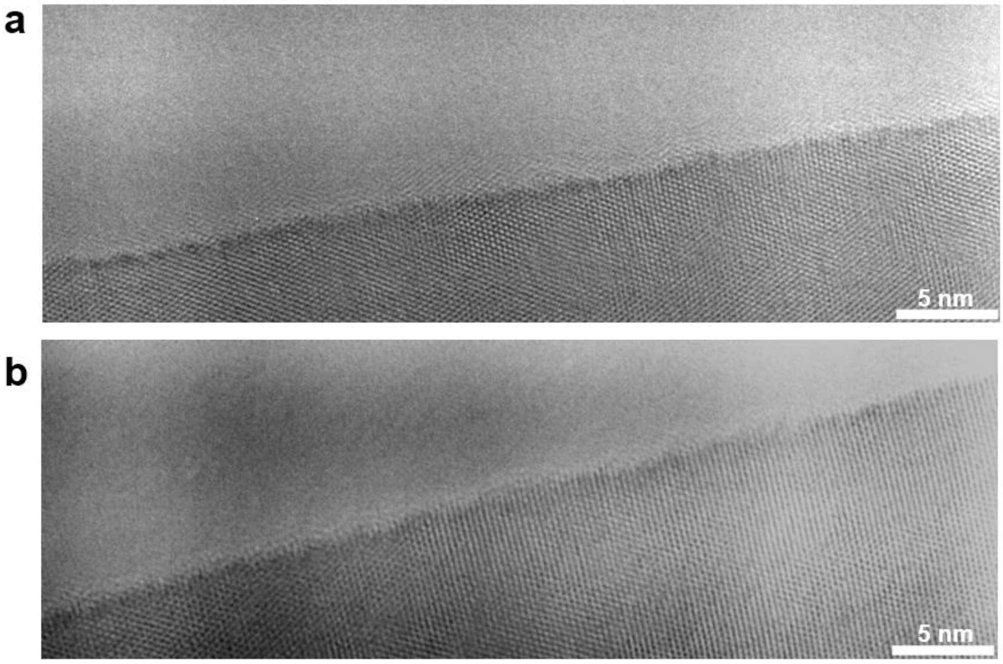

Supplementary Figure 23. HRTEM images of the edges of monolayer WS_2 single-crystal domains, showing armchair crystallographic orientation.

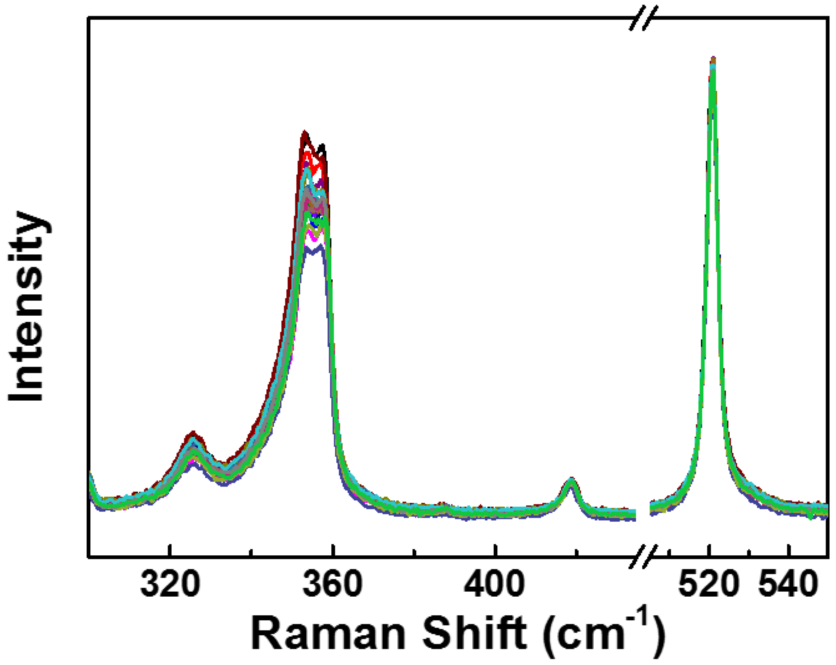

Supplementary Figure 24. Raman spectra taken from 20 random positions in the 400 μm single-crystal WS_2 domain in Fig. 1c, showing the formation of a uniform monolayer WS_2 domain.

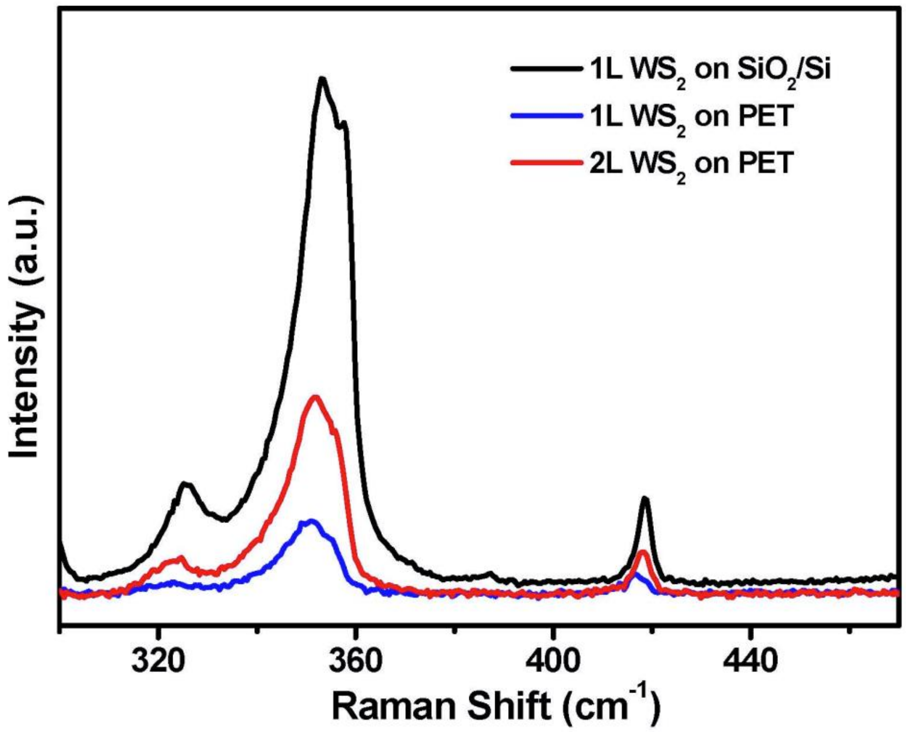

Supplementary Figure 25. Raman spectra of (blue plot) monolayer and (red plot) double-layer WS_2 films transferred onto PET by roll-to-roll/bubbling and (black plot) monolayer single-crystal WS_2 domains transferred onto SiO_2/Si substrate. A SiO_2/Si substrate was placed underneath the transparent PET to enhance the Raman signal of the monolayer and double-layer WS_2 films on PET during measurements. The Raman intensity of the double-layer WS_2 film on PET is about twice of that of the monolayer film on PET, but remarkably lower than that of monolayer single-crystal WS_2 domains on SiO_2/Si substrate. This is attributed to the significant enhancement because of the close contact with the SiO_2/Si substrate. However, it is worth noting that all these samples show Raman peaks with almost the same position and width, indicating the high quality of the monolayer and double-layer WS_2 films produced by the roll-to-roll/bubbling method.

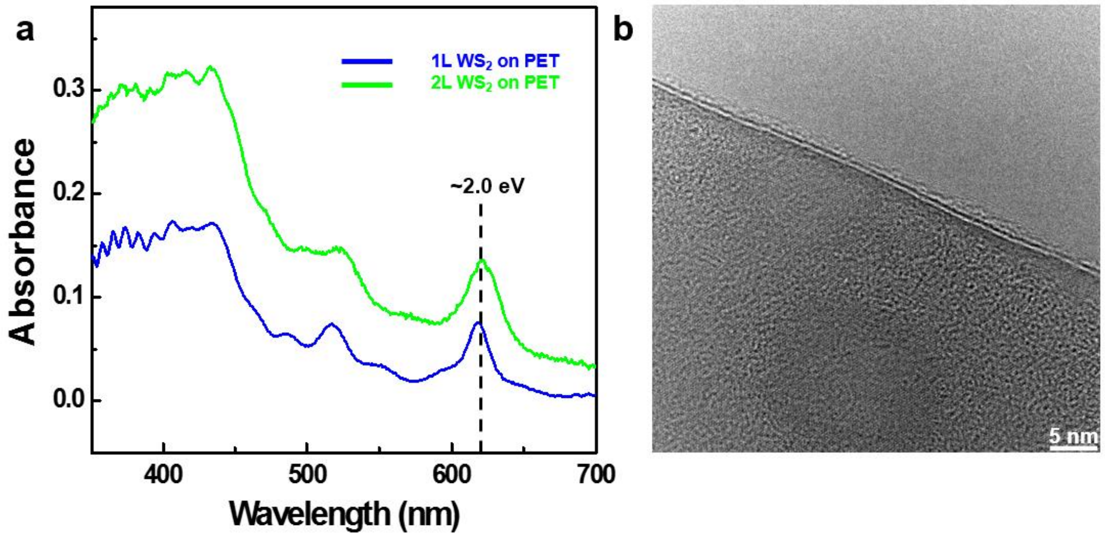

Supplementary Figure 26. (**a**) UV-visible absorption spectra of the WS_2 films transferred from one and two pieces of WS_2 films grown on Au foil onto a PET by roll-to-roll/bubbling. (**b**) HRTEM image of a WS_2 film, showing double-layer structure. This film was obtained by transferring two pieces of WS_2 films grown on Au foil onto the same SiO_2/Si substrate, followed by transferring onto a TEM grid by etching away the SiO_2/Si substrate.

Supplementary Note 1. Related result was published online[1] during the revision of this manuscript. As reported in Supplementary Ref. 1, the authors used ammonium metatungstate as tungsten precursor and H_2S as sulphur precursor to grow WS_2 on Au at a temperature of 935 ºC. Different from our observations of self-limited growth, additional multilayer WS_2 domains are formed and their coverage increases with extending the growth time after the growth of continuous monolayer WS_2 film. The authors also found that high growth temperature helps to increase the domain size of WS_2. At 865 ºC, only WS_2 domains with a size of < 30 μm are obtained, which is much smaller than the WS_2 domains obtained by our method at similar growth temperature (800 ºC), mostly larger than 500 μm. In addition, the authors also used electrochemical bubbling method to transfer the as-grown WS_2 on Au onto SiO_2/Si substrates. However, the transferred WS_2 films are incomplete with many empty portions and cracks, but show a higher mobility than our samples. As shown in our manuscript, the concentration of tungsten and sulphur precursors plays an important role in the controlled growth of monolayer WS_2. Therefore, the above differences are probably due to the different tungsten and sulphur precursors used and different growth parameters such as growth temperature.

Supplementary References

1 Yun, S. J. *et al.* Synthesis of centimeter-scale monolayer tungsten disulfide film on gold foils. *ACS Nano* **9**, 5510-5519 (2015).
